# Decontamination and Surface Analysis of PFAS-Contaminated Fire Suppression System Pipes: Effects of Cleaning Agents and Temperature

**DOI:** 10.1021/acs.est.4c09474

**Published:** 2025-01-23

**Authors:** Björn Bonnet, Matthew K. Sharpe, Gulaim Seisenbaeva, Leo W. Y. Yeung, Ian Ross, Lutz Ahrens

**Affiliations:** † Department of Aquatic Sciences and Assessment, Swedish University of Agricultural Sciences, Uppsala 75651, Sweden; ‡ Surrey Ion Beam Centre, University of Surrey, Guildford, Surrey GU2 7XH, U.K.; § Department of Molecular Sciences, Swedish University of Agricultural Sciences, Uppsala 75651, Sweden; ∥ SMTM Research Centre, School of Science and Technology, Örebro University, Örebro 70182, Sweden; ⊥ CDM Smith, 220 Montgomery Street. Suite 1418, San Francisco, California 94104 USA, United States

**Keywords:** Per- and polyfluoroalkyl substances, AFFF, foam transition, desorption, rebound effect, surfactant-surface interactions, supramolecular assemblies, butyl carbitol

## Abstract

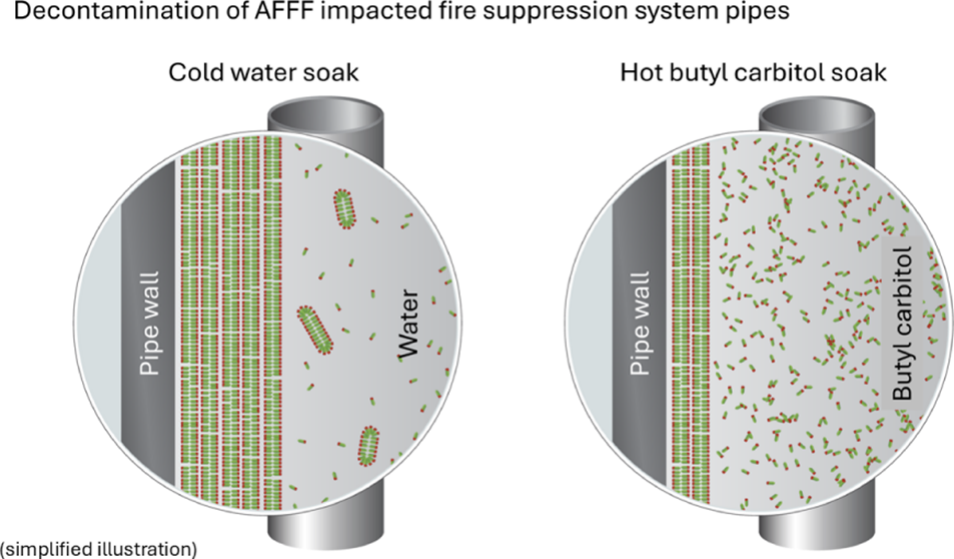

Per- and polyfluoroalkyl substances (PFAS)-containing firefighting foam have been used in stationary fire suppression systems for several decades. However, there is a lack of research on how to decontaminate PFAS-contaminated infrastructure and evaluate treatment efficiency. This study assessed the removal of PFAS from stainless steel pipe surfaces using different cleaning agents (tap water, methanol, and aqueous solutions containing 10 and 20 wt % of butyl carbitol (BC)) at different temperatures (20 °C, 40 °C, and 70 °C). The content of the remaining fluorine (F)-containing compounds on the pipe surfaces was evaluated for the first time using time-of-flight elastic recoil detection (ToF-ERD). The results showed that a 20% BC aqueous solution heated to 70 °C removed up to 40 μg/cm^2^ ∑PFAS from surfaces via soaking (targeted analysis). Treatment with 20% BC was 2- to 8-fold more effective than tap water at 70 °C and 10- to 20-fold more effective than tap water at 20 °C. Total fluorine analysis determined by combustion ion chromatography showed a 2- to 8-fold higher F-equivalent compared to targeted analysis in the cleaning solution after treatment, indicating the presence of a significant amount of polyfluoroalkyl PFAS. Surface analysis with ToF-ERD confirmed partial F removal from pipe surfaces throughout consecutive soaking intervals, with residual F remaining on pipe surfaces after treatment, leaving the risk of PFAS rebound into F-free firefighting foams. Furthermore, supramolecular assemblies of PFAS with at least 70 PFOS molecules/nm^2^ were identified by ToF-ERD on pipe interior surfaces.

## Abstract 2

Minimal research exists on AFFF-impacted fire suppression systems. This study reports a novel approach to decontaminate AFFF-impacted pipes and points out challenges in the assessment of successful decontamination.

## Introduction

Per- and polyfluoroalkyl substances (PFAS) are a group of synthetic chemicals that have some unique physical-chemical properties, such as chemical and thermal stability, resulting in extreme environmental persistence.^1−4^ This has led to ubiquitous detection in different environmental matrices^5−12^ and adverse health effects due to human exposure.^13^ In general, PFAS are characterized by containing perfluorinated carbons,^14^ which may form perfluoroalkyl chains of different lengths and may contain differing polar functional groups.^15^ PFAS mass production started in the 1930s^16^ and was subsequently applied in numerous industrial and consumer products,^17−20^ and fluorinated firefighting foams, such as fluoroprotein foams and aqueous film-forming foams (AFFFs).^1,21−25^ Different AFFF products contain a wide range of PFAS, such as legacy PFAS like perfluorooctanesulfonic acid (PFOS), perfluorooctanoic acid (PFOA), and novel polyfluoroalkyl precursors which may be zwitterionic, cationic, and anionic.^26−30^

Releases of fluorinated foams for extinguishment of Class B flammable liquid fires at airports, oil refineries, military bases, and during practice use at fire-training facilities are a major source of PFAS entering the environment.^6,21,22,28,29,31,32^ AFFF is also applied in stationary sprinkler systems, which consist of storage tanks for AFFF concentrate, foam proportioners, vast pipe networks, and sprinkler heads.^33^ Recurring release of fluorinated firefighting foams in suppression system testing or accidental discharge can lead to contamination of fire suppression infrastructure with PFAS.

Due to increasingly stringent regulatory guidelines being enacted, fluorinated firefighting foams are progressively being replaced with fluorine-free foams (FFF, which we refer to as F3 foams).^34−39^ The European Union (EU) published a regulation that will come into full force on 4th July, 2025, banning “C_8_” foams with regulatory limits of 25 μg/L for PFOA and 1,000 μg/L for PFOA precursors^34^ and “C_9_–C_14_” foams, with regulatory thresholds for ∑C_9_–C_14_ perfluorocarboxylic acids (PFCAs) at 25 μg/L and 260 μg/L for C_9_–C_14_ PFCA precursors.^40^ However, these guideline values are likely to be breached even by using F3 foam with old infrastructure, since PFAS may have adsorbed to the inner surfaces of the fire suppression infrastructure^33^ and will potentially leach out and get into F3 foams. PFAS concentrations of up to 1.6 g/L have been observed in F3 foams without sufficient decontamination of fire suppression systems.^41^

Decontamination procedures and techniques on fire suppression infrastructure (e.g., pipework, steel and synthetic storage tanks, hoses) have been reported in peer-reviewed literature,^33,42,43^ nonpeer- reviewed literature, technical reports,^44−47^ and webinars.^48^ Multiple cleaning agents, such as tap water (TAP), TAP-solvent mixtures with other additives, glycols, and proprietary commercially available products, were tested for their potential PFAS removal from contaminated infrastructure. In general, PFAS removal was higher for solvent- or glycol-based solutions and proprietary products as compared to TAP.^42−48^ Adjustment of pH and increased temperature also showed positive effects on PFAS removal.^43^ When removing PFAS from the walls of fire suppression system infrastructure, one issue encountered is described as the rebound effect.^43,47^ This phenomenon refers to the observed increase in PFAS concentrations in cleaning agents, TAP, or F3 foam after an initial PFAS reduction during reagent flushes used for decontamination. This rebound effect demonstrates that a significant mass of residual PFAS remains associated with the interior surfaces of fire suppression infrastructure and that a retention mechanism that promotes surface storage of PFAS must exist. The formation of supramolecular structures formed by amphiphilic PFAS (fluorosurfactants) has been described in several articles in physical chemistry journals, which describe their self-organization into multilayered membranes with enhanced stability.^49,50^ Removal of these structures is essential to confirm successful decontamination and minimize PFAS rebound before switching to F3 foams. To date, little is known about the effectiveness of cleaning agents with respect to the remaining PFAS mass on sprinkler system pipes after treatment.

Despite previous advances in the decontamination of fire suppression infrastructure, there are limitations associated with the techniques outlined above. For example, the use of proprietary cleaning agents is limited by a lack of discussion and understanding of the mechanism regarding the solvation of PFAS assemblies. Furthermore, chemical analysis of PFAS concentration in liquid cleaning agents used for decontamination does not consider the residual mass of PFAS that may remain on the pipework surfaces, resulting in insufficient evidence to confirm effective decontamination. Therefore, credible chemical analytical methods are required to assess the PFAS concentrations on the inner surfaces of fire suppression systems for coverage of PFAS.

In this study, we aim to investigate the effectiveness of butyl carbitol (BC) (CAS number: 112–34–5, Merck, Germany), commonly used as an effective stabilizing solvent for PFAS^23,25^ in AFFF formulations, at concentrations of 10 and 20 mass% (m.%) in aqueous solution. The performance of BC was compared to that of TAP and methanol (MeOH) in removing adsorbed PFAS from AFFF-contaminated sprinkler system pipes. We also assessed the effect of temperature elevations in incubation experiments at 20 °C, 40 °C, and 70 °C. Furthermore, we conducted a quantitative analysis of the surfaces and approximately the upper 200 nm of AFFF-impacted stainless steel pipes, assessing their elemental concentration before, during, and after treatment by using time-of-flight elastic recoil detection (ToF-ERD) to identify any remaining PFAS mass on the pipe surfaces.

## Material and Methods

### Selection of AFFF-Contaminated Pipe Sections

The pipe sections used in this experiment were decommissioned stainless steel (316L) fire suppression system pipes from a large industrial production site in Uppsala, Sweden. The fire suppression system comprises a vast network across the entire company premises. Decommissioning took place in multiple sections, but there is no record of where the pipes are from in the system. The pipe diameter range (3.9–5.7 cm) supports the conclusion that they were positioned on the foam side rather than the AFFF concentrate side of the fire suppression system. The pipes were in use with PFAS-containing AFFF for two to three decades. The usage of four different AFFF concentrates, produced by electrochemical fluorination (ECF) and fluorotelomer (FT)-based products, has been documented. Furthermore, several releases of AFFF in different parts of the fire suppression system have occurred and might have contributed to different PFAS loadings in both concentration and composition. After ultrasonication-supported extraction using MeOH of nine pipe sections (A–J), three pipe sections with the highest PFAS levels were selected for further experiments (F, H, and I). The three pipe sections were characterized using scanning electron microscopy (SEM) (for details see Text S1, Figure S1, and Table S1).

### Experimental Design—Soaking Experiment

**Figure 1:**
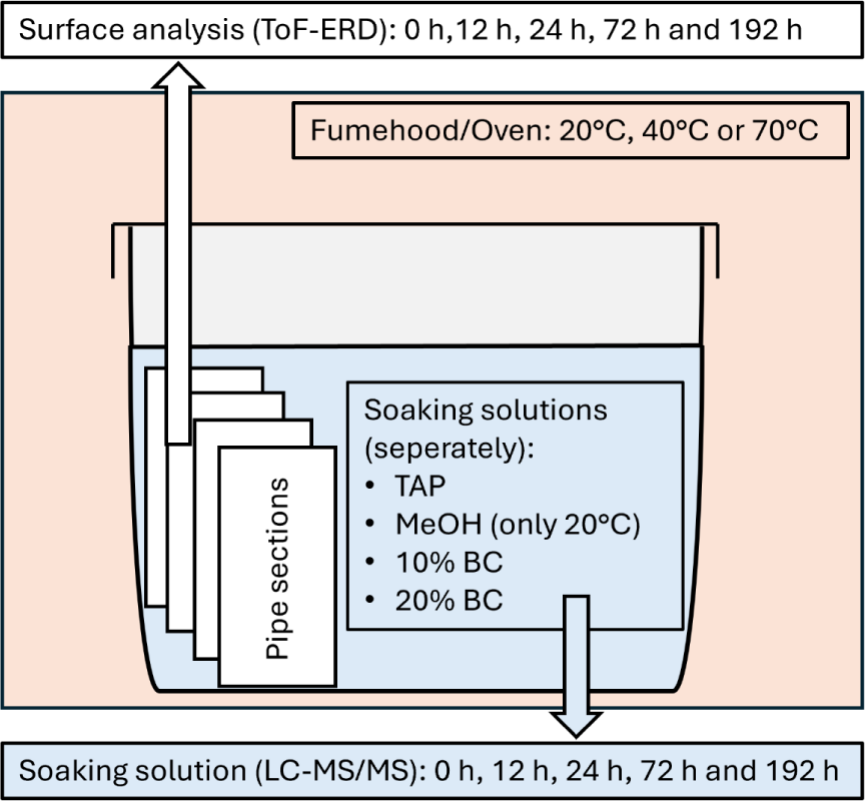
Illustration of the experimental design of the cleaning solution soaking experiment.

In the soaking experiment, pipe sections were incubated in 500 and 1000 mL polypropylene (PP) containers filled with four different cleaning solutions separately, and they were tested at three different temperatures (room temperature (20 °C), 40°C, and 70 °C) (Figure 1). The four different cleaning solutions were: (i) pure TAP, (ii) TAP containing 10 mass% BC (BC10), (iii) TAP containing 20 mass% BC (BC20), and (iv) pure MeOH (LiChrosolv, hypergrade for LC-MS, Merck, Germany). MeOH was only used at 20 °C. For this, pipes were cut into similarly sized quarters using a metal bandsaw (Meec Tools, Metal bandsaw 230 V, 1100 W) and angle grinder (Makita, DGA521, 18 V). Due to different dimensions in length (60 cm −100 cm) and diameter (3.9 cm −5.7 cm) of the initial pipe, the pipe sections used for the soaking experiment differed in size (38–68 cm^2^) and, therefore, in AFFF-contaminated area as well (Text S2 and Table S2). Importantly, only pipe sections of the same initial pipe were put together in a PP container. Pipe sections of each pipe were prepared for different combinations of treatment solution and temperature scenarios, representing experimental triplicates (Table S2). After 12 h of the experiment, the pipe sections were removed from the container and put into another container filled with a fresh soaking solution of the same kind, volume, and temperature. This exchange was repeated at 24 and 72 h after the start of the experiment. After 8 days of soaking, the experiment was stopped by removing all pipe sections from their containers. This yielded a total of five time points (0 h, 12 h, 24 h, 72 h, and 192 h) for each pipe section, soaking solution, and temperature. PFAS concentration was determined in the aqueous solution for each time point separately. A rebound experiment was performed after the 8-day soaking experiment. Pipe sections H and I were air-dried and put into 1 L polyethylene freezer zip bags, and stored in darkness for 4 months at 20 °C. Subsequently, the pipe sections were individually incubated in TAP for 7 days at 20 °C, and TAP was analyzed for PFAS.

### PFAS Target Analysis

A total of 24 PFAS (Text S3) were analyzed, including 11 C_3_–C_13_ PFCAs (PFBA, PFPeA, PFHxA, PFHpA, PFOA, PFNA, PFDA, PFUnDA, PFDoDA, PFTriDa, PFTeDA), seven C_4_–C_10_ PFSAs (PFBS, PFPeS, PFHxS, PFHpS, PFOS, PFNS, PFDS), three FT-sulfonates (4:2 FTSA, 6:2 FTSA, and 8:2 FTSA), *N*-methyl- and ethyl-perfluorooctane sulfonamido acetic acid (Me-FOSAA, Et-FOSAA), and perfluorooctane sulfonamide (FOSA). Nineteen mass-labeled internal standards (IS) were used (Wellington Laboratories MPFAC-24 mixture), including, ^13^C_4_–PFBA, ^13^C_5_–PFPeA, ^13^C_5_–PFHxA, ^13^C_4_–PFHpA, ^13^C_8_–PFOA, ^13^C_9_–PFNA, ^13^C_6_–PFDA, ^13^C_7_–PFUnDA, ^13^C_3_–PFDoDA, ^13^C_2_–PFTeDA, ^13^C_3_–PFBS, ^13^C_3_–PFHxS, ^13^C_8_–PFOS, ^13^C_2_–4:2 FTSA, ^13^C_2_–6:2 FTSA, ^13^C_2_–8:2 FTSA, ^13^C_8_–FOSA, D_3_–MeFOSAA, and D_5–_EtFOSA.

All samples from the soaking experiments were prepared for direct injection analysis by ultraperformance liquid chromatography coupled to tandem mass spectrometry (UPLC-MS/MS) analysis (Sciex Triple Quad 3500 LC-MS/MS, USA) (for details, see Text in S3 and Smith et al).^51^ Limits of detection and quantification, as well as method recoveries, are presented in Tables S3 and S4.

### Total Oxidizable Precursor (TOP) Assay

To get better estimates of total PFAS concentration in the samples, a TOP assay was performed for samples of the first soaking interval (12 h) on all pipe sections (F, H, and I). Due to the inhibition of PFAS oxidation in the presence of BC, samples containing BC were not included for the TOP assay (for details, see Text S4 and Tables S5–S7). TOP assays were performed in accordance with the originally proposed conditions (60 mM K_2_O_8_S_2_/150 mM NaOH).^52^ MeOH samples were evaporated to dryness under an N-stream and reconstituted in 1 mL of Milli-Q-water. The volume of sample, oxidant, base, and acid neutralization, as well as pH measurements throughout oxidation, are reported in Tables S8 and S9.

### Total Fluorine (TF) Analysis

Due to the inhibition of PFAS precursor oxidation in the TOP assay by BC (see Text S6), total fluorine (TF) analysis was performed to get an insight into how much unrecognized PFAS is present in the samples using a combustion ion chromatography (CIC) system (for details, see Sections S6 and S7) for future experiments. Pipe section I was selected based on the results of the target analysis that showed the highest PFAS concentrations in the first time interval.

### Surface Analysis with Time-of-Flight Elastic Recoil Detection Analysis (ToF-ERD)

ToF-ERD analysis was used for elemental analysis of surfaces.^53^ These data provide accurate measurements for every element that is present on the surface to depths between 100 and 200 nm. The elements of interest for our investigations were carbon (C) and fluorine (F), as these two elements are the predominant elements in PFAS molecules, and iron (Fe), which is the major component of stainless steel (for details, see Section S8 and Figure S2). ToF-ERD measurements were done on pipe section H.

### Statistical Analysis

A repeated measures ANOVA was performed using time points (12, 24, 72, and 192 h), temperatures (20 °C, 40 °C, and 70 °C), and treatment solutions (TAP, BC10, and BC20) as fixed factors, including all interactions. MeOH treatment was not considered since it was used only within the 20 °C scenario. Differences were checked for the treatment solutions and temperatures. The statistical analysis was performed in R version 4.3.2.^54^

## Results and Discussion

### Kinetics of PFAS Removal and Rebound Effect

PFAS desorption occurred predominantly within the initial soaking interval of 12 h, removing, on average, 68% ± 22% (minimum average: 40% for TAP 20 °C; maximum average: 99% for MeOH 20 °C) with respect to the ∑_24_PFAS after 192 h. Additional desorption of ∑_24_PFAS in the following time intervals was limited to 13% ± 8% (24 h), 11% ± 8% (72 h), and 8% ± 8% (192 h) (see also Figure S3 and Table S10). PFOS was the most abundant single compound measured in the soaking solutions, with an average of 76% ± 22% of ∑_24_PFAS, followed by PFOA (10% ± 14%), 6:2 FTSA (6% ± 8%), PFHxS (5% ± 5%), 8:2 FTSA (3% ± 5%), and PFHxA (2% ± 2%) across all treatment solutions (TAP, MeOH, BC10, and BC20) and temperatures (20 °C, 40 °C, and 70 °C). The high contribution of PFOS indicates that the sprinkler system pipes used in our experiment were predominantly impacted by 3M AFFF formulations (e.g., 3M Light Water).^55^ The presence of 6:2 FTSA and unknown precursors suggests that the pipes were also impacted by FT-based AFFF formulations.^26,55^ Desorption of PFAS generally followed chain length and headgroup-dependent trends (for details, see Sections S10, S11 and Figures S4 and S5).

The 7-day rebound test using TAP (see Section S12 and Figure S5) showed that in most cases, ∑PFAS concentrations in rebound TAP were higher than in the respective soaking solution after 192 h during the soaking experiment. Furthermore, ∑PFAS concentrations in the rebound test were lower for BC20 solutions and scenarios at 70 °C compared to TAP and BC10 at 20 and 40 °C. In the case of TAP (20 °C), ∑PFAS concentrations in the rebound test were in the same range as they were in the initial soaking interval (12 h). The rebound experiment showed that the continuous drop of ∑PFAS concentrations throughout the soaking experiment did not reflect complete PFAS removal from the pipe surfaces and that comparisons between high ∑PFAS concentrations in the initial cleaning step to low(er) ∑PFAS concentrations in following cleaning steps are not a plausible way to demonstrate successful decontamination. Credible rebound tests, in which a previously purified system was exposed to TAP or F3 foam for numerous days, have only been investigated in a few studies. Lang and Devine^46^ found PFAS rebound into TAP and F3 foam during a 3-day exposure following a final short-term water flush in which no PFAS were detected. Dahlbom et al.^43^ assessed PFAS rebound into TAP after decontamination was performed and found gradually increasing PFAS concentrations over a period of 157 days. Accordingly, Nguyen et al.^47^ performed a 6-week rebound experiment using TAP following decontamination and observed steadily increasing PFAS concentrations.

### Effect of Temperature on Removal of PFAS

**Figure 2:**
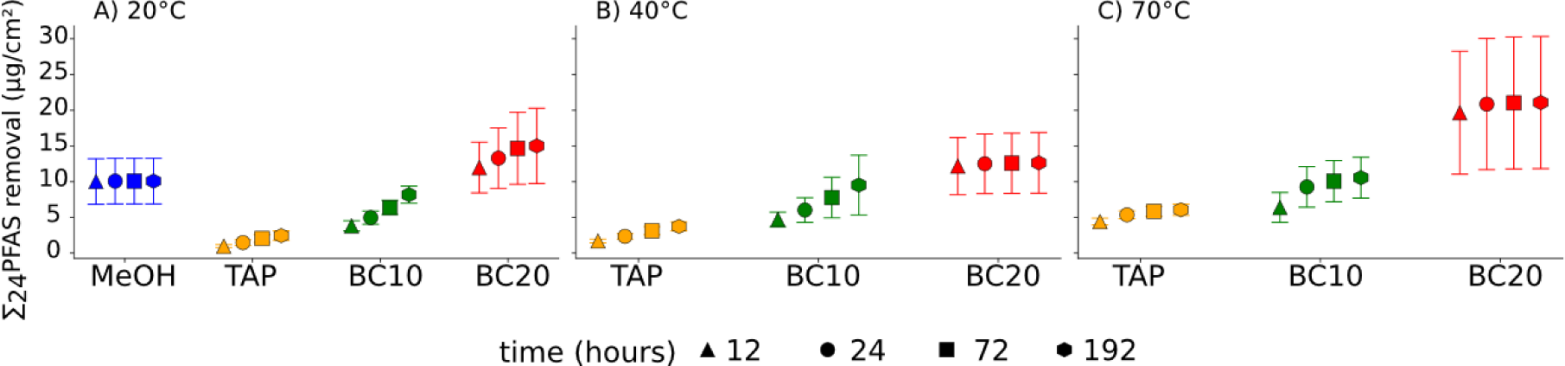
PFAS removal (μg/cm^2^) from stainless steel pipes using methanol (MeOH) (only 20 °C), tap water (TAP), 10 wt % butyl carbitol in TAP (BC10), and 20 wt % BC in TAP (BC20), respectively, during soaking experiments at A) 20 °C, B) 40 °C, and C) 70 °C. Data points represent average concentrations (*n* = 3), with standard error as error bars.

Effects of temperature on PFAS removal are shown in Figure 2 for the average values for three independent pipe sections (for single pipe sections, see Section S12 and Figure S5). Increased temperature generally increased the removal efficiency of PFAS for the same treatment solution steadily. For TAP, the ∑_24_PFAS removal increased by 150% from 2.4 μg/cm^2^ (20 °C) to 6 μg/cm^2^ (70 °C) at 192 h. Statistically significant differences were observed for TAP (20 °C) and TAP (70 °C) at 12 h (*p* < 0.05) and 24 h (*p* < 0.05). For BC10, the ∑_24_PFAS removal increased by 30% from 8.1 μg/cm^2^ (20 °C) to 10.5 μg/cm^2^ (70 °C) at 192 h. For BC20, the ∑_24_PFAS removal increased by 40% from 15 μg/cm^2^ (20 °C) to 21 μg/cm^2^ (70 °C) at 192 h. Accumulated PFAS concentration for BC20 (40 °C) was 16% lower than for BC20 (20 °C) after 192 h, which can be related to measurement uncertainty and heterogeneous distribution of PFAS on pipe sections. For MeOH, only 20 °C was tested showing a ∑_24_PFAS removal of 10 μg/cm^2^ at 192 h. Increased temperature showed increasing solubility of PFAS assemblies from surfaces into solution, which indicates that supramolecular aggregates were solubilized more efficiently into smaller structures and monomers with increasing temperatures. Below the Krafft-point *T*_*K*_ and at sufficiently high surfactant concentrations, surfactants will neither be present as micelles nor as monomers but assemble in various crystalline aggregates, e.g., bilayers.^56^ Above *T*_*K*_, surfactants’ solubility increases, and aggregates “melt” into monomers below the critical micelle concentration (CMC) or micelles above CMC. Our results align well with this since higher PFAS concentrations were observed in solutions at elevated temperatures. For Na-PFOS, up to 75 °C and 8.5 mmol/L for *T*_*K*_ and CMC, respectively, have been reported.^57^ Temperatures in our experiments ranged within and close to possible *T*_*K*_ for PFOS-based surfactants; however, concentrations measured in our solutions were well below the CMCs (0.02 mmol/L for Na-PFOS),^57^ and thus *T*_*K*_ has not been reached and micelle formation in solution did not occur. A similar observation was made previously,^58^ where single-chain perfluoroalkyl surfactants formed multilamellar and multilayered vesicles of several hundred nm in size at close to ambient temperature. However, at 40 °C, the structures were broken down into much smaller vesicles between 30–100 nm, and at 70 °C, into globules of 100 nm and fibers of 1–10 nm. Our measurements align to some extent with a previous study,^33^ which tested a commercially available and proprietary product for the decontamination of fire suppression systems (Fluoro Fighter, FF) at temperatures of 22 °C, 40 °C, and 80 °C. Their results showed that PFAS were removed from stainless steel pipes into solution using FF and ∑PFAS concentrations increased from ∼4 μg/cm^2^ to ∼6 μg/cm^2^ between 22 and 40 °C, whereas they decreased from 6 μg/cm^2^ to 5 μg/cm^2^ between 40 and 80 °C. Lower removals at 80 °C were attributed to heterogeneity in PFAS distribution on pipe surfaces. Dahlbom et al.^43^ compared the removal of PFAS at 22 and 50 °C for a cleaning solution consisting of 44.9% MQ water, 44.9% isopropanol (IPA), and 0.2% sodium hydroxide (25 wt % in MQ water) and found that PFAS removal was slightly higher at 50 °C (∼23 μg/cm^2^) compared to 22 °C (∼19 μg/cm^2^) for galvanized steel but lower at 50 °C (∼195 ng/cm^2^) compared to 20 °C (∼220 ng/cm^2^) for stainless steel. Nguyen et al.^47^ tested TAP, a solution containing TAP, propylene glycol (20%), ethanol (10%), and acetic acid (2%) (CSM solution), and a proprietary cleaning agent at 22 and 50 °C in flow-through experiments on 304 stainless steel pipes. In the flow-through experiments, they found that heating increased PFAS removal from 160 to 240 ng/cm^2^, from 250 to 360 ng/cm^2^, and from 290 to 450 ng/cm^2^ for the proprietary solution, TAP, and CSM solution, respectively. Temperature effects in the present study were smaller between 20 and 40 °C than they are between 40 and 70 °C, which suggests that temperatures near *T_K_* should be pursued for the most optimal conditions for PFAS removal.

### Comparison of Cleaning Solutions for Removal of PFAS

In general, the removal of ∑_24_PFAS on the pipe surfaces increased for the solutions in the following order: TAP < BC10 (<MeOH) < BC20. The average increase between TAP and BC10, and between BC10 and BC20, accounted for 154% ± 66% and 71% ± 29%, respectively (Figure 2). We observed statistically significant differences for ∑_24_PFAS between TAP and BC20 at 20 °C (12 h: *p* < 0.0005; 24 h: *p* < 0.001; 72 h: *p* < 0.005; 192 h: *p* < 0.005), at 40 °C (12 h: *p* < 0.005; 24 h: *p* < 0.05; 72 h: *p* < 0.05), and at 70 °C (12 h: *p* < 0.05). Further statistically significant differences were observed for TAP and BC10 at 20 °C (12 h: *p* < 0.05; 24 h: *p* < 0.05; 192 h: *p* < 0.05).

The dissolution effects for surfactants in the presence of pure alcohol or due to the addition of alcohol to an aqueous solution are related to the ability of alcohols to increase the surface activity and van der Waals forces between surfactant molecules, thus lowering the CMC of surfactants.^59^ The longer the chain length of the alcohol, the larger the decrease of the CMC.^60,61^ The decrease of CMC has also been shown to be dependent on the number and type of polar groups associated with the alcohol.^62^ Both effects of chain length and polar groups within the alcohol molecule can explain why BC is more effective at 20% concentration than pure MeOH, due to the longer molecular chain and more polar sites within the molecule. Further confirmation of this can be concluded from the log *n*-octanol–water partition coefficient (*K*_OW_) which is lower for MeOH (*K*_OW_ = −0.77) compared to BC (log *K*_OW_ = 0.56), indicating a higher affinity of PFAS with BC. Dong et al.^63^ demonstrated a CMC for PFOA in an aqueous solution of 26.5 mM, which was reduced to 14.2 mM and 13 mM with 10% and 20% addition of ethanol, respectively. The large initial reduction of CMC for 10% ethanol in water was attributed to the cosolubilization of ethanol molecules into the PFOA micelle, resulting in reduced surface charge density and lower headgroup repulsion at the micelle surface (cosurfactant effect). On the other hand, the lower reduction of CMC between 10% and 20% addition of ethanol in water was explained by the cosolvent effect, which, in addition to the cosurfactant effect, is influenced by increases in CMC due to disruption of the water structure network, resulting in a reduction in the hydrophobic effect, and thereby the micelle size and intermicellar distance between micelles decrease. As mentioned in the previous chapter, “Effects of temperature on removal of PFAS”, it is not suggested that micelle formation is taking place in solution; however, it is suggested that factors leading to the reduction of micelle size will contribute to dissolve formations on surfaces. Dong et al.^63^ observed a reduction in micelle size by 34% and 55% in the presence of 10% and 20% ethanol (EtOH) in aqueous solution, respectively, which could promote the disruption of PFAS assemblies on the pipe surfaces and their subsequent dissolution. Giles et al.^64^ reported similar effects for 6:2 FT-sulfonamide alkylbetaine (6:2 FTAB), a common constituent in FT-based AFFF formulations, in the presence of up to 0.5 wt % BC. They found 6:2 FTAB micelles to decrease in size in the presence of BC and reasoned that BC was incorporated into the 6:2 FTAB micelles’ palisade layer. Similarly, as explained above for MeOH, BC is considered to have a higher potential for preventing PFAS aggregation than EtOH. In fact, BC is a major constituent in most AFFF formulations, serving as a solvent for fluorosurfactants and hydrogen surfactants to allow for storage stability and improved shelf life of AFFF concentrate.^23,25,65^

### Comparison of Target PFAS Analysis with TOP Assay and TF

**Figure 3:**
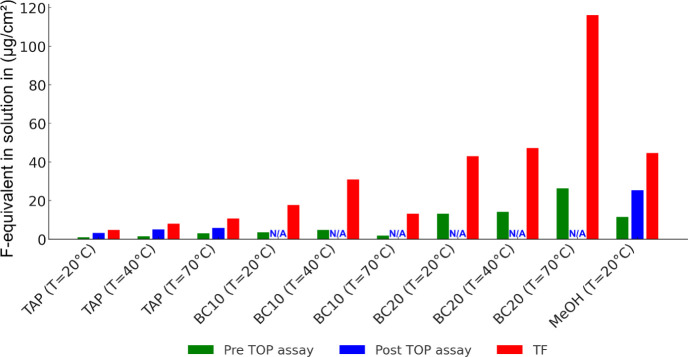
F-equivalent concentrations for target analysis before the TOP assay (green) and after the TOP assay (blue) via LC-MS and total fluorine (TF) analysis via CIC (red). Measurements only for pipe I. NA = not available.

PFAS targeted analysis before the TOP assay was compared to targeted analysis after the TOP assay and TF (Figure 3). The reported F-equivalent concentrations are shown in the following order: targeted analysis before the TOP assay < targeted analysis after the TOP assay < TF. F-equivalent concentrations increased by a factor of 2–4 between targeted analysis before the TOP assay and targeted analysis after the TOP assay and by a factor of 2–8 between targeted analysis before the TOP assay and TF analysis. As shown in previous studies, ∑PFAS concentrations increased during the TOP assay due to the oxidation of unknown precursor PFAS,^52^ which is further confirmed by the increased relative contribution of short-chain PFCAs (<C_8_) between targeted analysis before the TOP assay (2% ± 3%) compared to targeted analysis after the TOP assay (46% ± 13%) (Section S12 and Figure S6).

The observed differences between the TOP assay and TF analysis could be related to factors, such as incomplete oxidation of precursor PFAS during the TOP assay,^66^ incomplete recovery of long-chain PFCAs and PFSAs after oxidation^52,67−69^ and the formation of ultrashort-chain PFCAs during oxidation.^69−72^ For example, Patch et al.^70^ have shown that perfluoropropionic acid (PFPrA) could account for up to 19% of ∑PFAS. Previous studies have also shown that the presence of other organic substances, which are present in AFFF, can inhibit PFAS oxidation,^73^ as this study has shown for BC (see Table S6 and Text S4). The TOP assay experimental setup in this study does not allow for the correction of incomplete recoveries of long-chain PFCAs and PFSAs or other compounds, since no mass-labeled surrogates were introduced before oxidation.^70^ Decomposition/mineralization of PFCAs by sulfate radicals due to decreasing pH^74^ can be ruled out as the pH remained high (pH = 14) after oxidation (Table S9).

### Surface Analysis of Sprinkler System Pipes by ToF-ERD

**Figure 4:**
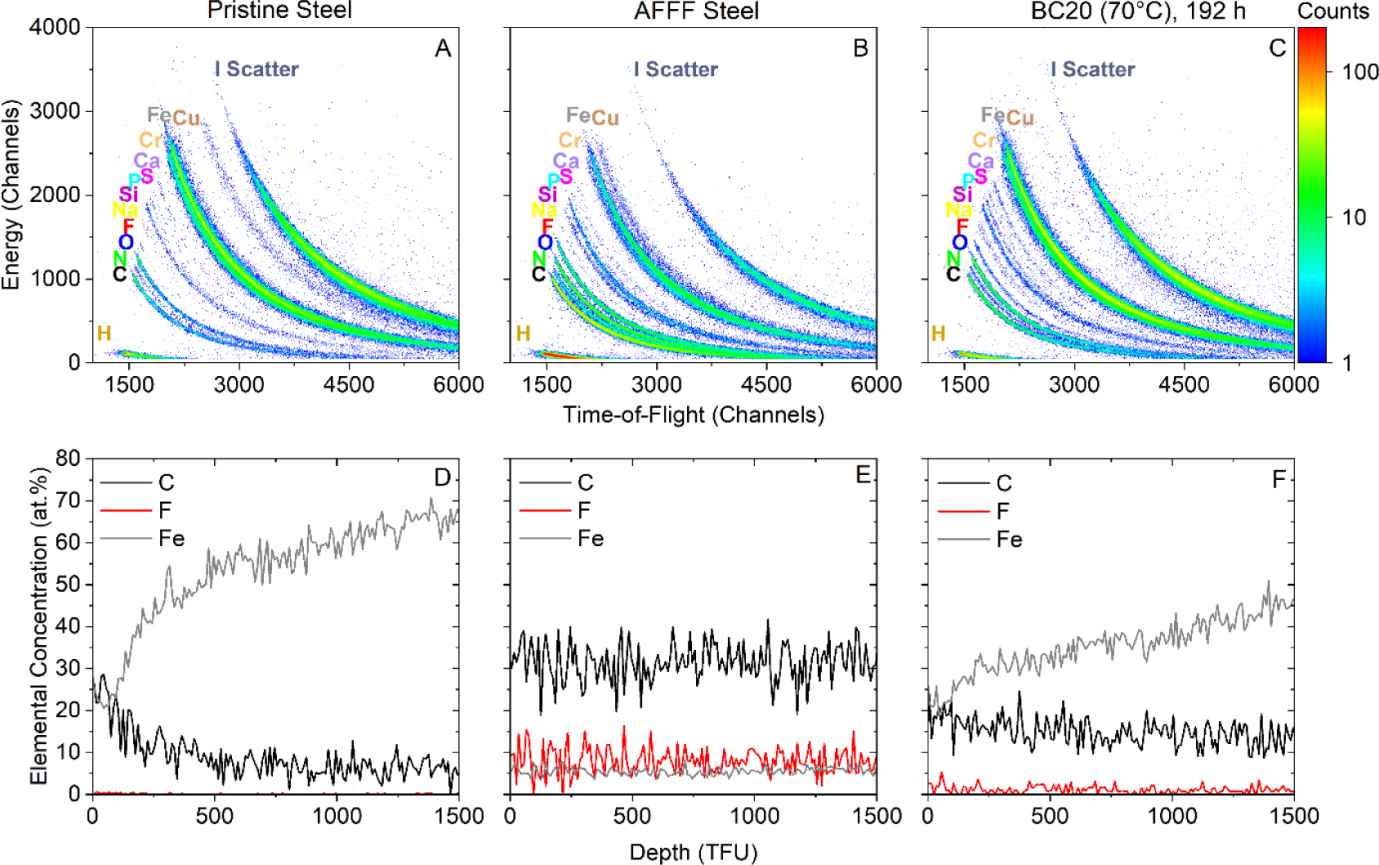
ToF-ERD histograms (top) and depth charts (bottom) for (A) and (D) exterior pipe (hereafter referred to as pristine stainless steel), (B,E) AFFF-impacted stainless steel pipe, and (C,F) pipe treated with BC20 at 70 °C after 192 h. Histograms represent the total elemental composition of the pipe surfaces. Curved plots are derived from velocity (*x*-axis) and energy (*y*-axis) measurements. Depth profiles show a selection of carbon (C), fluorine (F), and iron (Fe). Depth profiles indicate elemental concentrations (*y*-axis) with respect to the analytical depth (*x*-axis) of the ion beam (for details, see Text S13).

Surface analysis with ToF-ERD revealed interesting trends in F, C, and Fe as well as depth profiles between pristine steel, untreated AFFF-impacted pipe sections, and pipe sections after treatment (Figure 4, Section S13,Table S11 and Figures S7–S16). Despite the substantial PFAS removal from pipe sections by the most effective solution tested herein, BC20 70 °C, residual F remained on the pipe sections (Figure 4 C,F). After treatment by BC20 70 °C, F concentration was measured at 1.1 atomic % (hereafter at. %), which represents the lowest residual F concentration measured on pipe sections after treatment. The F concentration was consistently low within the depth profile (Figure 4F), whereas the Fe concentration increased from 20% at the surface (0 thin film units (TFU); representing an aerial unit of 10^15^ atoms per cm^2^) to 45% at 1500 TFU. This means that the Fe was no longer covered as much by an AFFF layer compared to the untreated AFFF-impacted pipe. C concentration was relatively constant in the depth profile (on average 15 at. %), decreasing from 20 to 15 at. % at 1500 TFU for the BC20 (70 °C) treated pipes. For pristine stainless steel pipe (Figure 4 A,D), the F concentration ranged around the measurement detection limit of 0.1 atomic %, while Fe and C were measured with average concentrations of 52 at. % and 9.3 at. %, respectively. In the depth profile of the pristine stainless steel pipe, the Fe concentration increased from 20 to 65 at. % (average concentration of 52 at. %) and the C concentration decreased from 25 to 5 at. % (average concentration of 9 at. %) until the analytical depth limit of 1500 TFU. In the AFFF-contaminated untreated pipe sections (*n* = 5) (Figure 4B,E), the average F concentration was 4.8 at. % (3.4–8.0 at. %), while average concentrations for C and Fe were 28 at. % (25–32 at. %) and 12 at. % (5.5–20.7 at. %), respectively. The depth profile showed consistent detection of all three elements throughout the entire analytical depth. The differences in concentrations and depth profiling between the pristine and AFFF-impacted pipe were a result of a layer composed of PFAS covering the pipe surface. Detection of both F and C indicates the presence of fluorinated carbons on the AFFF-impacted pipe. The lower detection of Fe on the AFFF-impacted pipe compared to that of the pristine stainless steel pipe indicates that the Fe within the pipe was covered and thereby shielded from being detected at a higher intensity as for the pristine stainless steel pipe. Furthermore, the constant detected rates in the histograms and the depth profiles indicate that the AFFF layer is thicker than the analytical depth of the ion beam (1500 TFU).

Further results of F measurements on the treated pipes at 70 °C were consistent with results from the soaking experiment, showing decreasing F concentration remaining on pipe surfaces after 192 h, with 1.8 at. % for TAP (70 °C) and 1.7 at. % for BC10 (70 °C). Regarding the results at 20 and 40 °C, this trend did not strictly uphold for measurements at 40 and 20 °C (Section S13). Despite increasing F removal during soaking, measurements of F, C, and Fe concentrations throughout every treatment interval did not show a consistent stepwise decrease (F and C) or increase (Fe) between the initially measured concentrations on pipe sections and the concentrations in the consecutive time intervals (Table S12). Incoherent behavior in the concentration of all three elements analyzed is related to a nonhomogeneously and nonuniformly distributed AFFF layer on the initial pipe.

However, for all pipe sections after 192 h of treatment, F was detected on the pipe surface with an average of 2.1 at. % for TAP, 2.8 at. % for BC10, 2.2 at. % for MeOH, and 1.8 at. % for BC20, indicating that even after the most efficient treatment (BC20 70 °C), there was still an F-containing layer left beyond the analytical depth of the ion beam. Partial removal of the AFFF-associated layer was indicated not only by decreasing F detection but also from the Fe and C depth profiles comparing treated and untreated pipes (Figure 4). There was a statistically significant decreasing trend for F (*p* < 0.0001) and C (*p* < 0.0001) with increasing Fe concentration (Figure S17). Comparisons of F measurements on surfaces before and after 192 h of treatment allow one to estimate total F removal efficiencies. Based on the average F concentration (4.8 at. %) for five measurements of untreated pipe sections, the highest F removal efficiency was achieved for BC20 (70 °C) with 77 at. % total F removal, followed by 65 and 63 at. % for BC10 (70 °C) and TAP (70 °C). Due to the AFFF-associated layer still being present beyond the analytical depth, the reported F removal efficiencies are likely to be an overestimation.

Previous efforts by Lang et al.^33^ identified PFAS assemblies on the pipe surfaces using scanning electron microscopy (SEM) and X-ray photoelectron spectroscopy (XPS) and revealed lower concentrations of elemental F on surfaces after treatment with FF (3 at. %–5 at. %) compared to TAP (7 at. %–17 at. %). However, SEM-XPS is very surface-sensitive, penetrating only the surface to a depth of 7–10 nm, and it cannot detect hydrogen (H). Therefore, the reported elemental concentrations should be seen as indicative. Dahlbom et al.^43^ performed surface analysis by SEM electron-dispersive X-ray spectroscopy (EDX) and showed reductions of F-containing structures during treatment without further quantification. SEM-EDX has a penetration depth in the low μm range, which exceeds the AFFF-associated layer deep into the stainless steel. Elemental compositions are therefore skewed toward the elements of steel. Measurements of F are therefore considered semiquantitative.^43,75^ ToF-ERD analysis surpasses the limitations regarding analytical depth, depth resolution, and incomplete elemental detection. The measurements by ToF-ERD are highly quantitative across the entire analyzed surface area of 12 mm^2^ (Figure S18). The precision is highlighted by the calculation of the number of detected atoms of each element, which allows to draw conclusions about the structural properties of the AFFF-associated layer.

### Measurements of PFAS Supramolecular Assemblies

Comparing the SEM image of the pipe exterior to the interior (Figure S1) reveals that the pipe interiors are coated with an amorphous solid mass (AFFF layer), while unexposed pipe surfaces comprise a flat cellular network that is common when imaging stainless steel.^76^ Even though the SEM visualization does not reveal any precise depth measurements, differences in both bright and dark areas on the images indicate large differences in layer depth qualitatively, confirming the hypothesis of a nonhomogeneously and nonuniformly distributed AFFF layer across the pipe surfaces. Measurements of F using ToF-ERD provide evidence that these amorphous structures on the pipe interior appear to be supramolecular aggregates of PFAS because ToF-ERD measurements allow a quantitative estimation of the number of F atoms/nm^2^ of the pipe surface within the measurement depth of 1500 TFU.

The estimated number of F atoms/nm^2^ (see Section S16) for the untreated AFFF-impacted surface is 1200, whereas the number of F atoms/cm^2^ for 20% BC (70 °C) is 163. Considering the AFFF layer mainly consists of PFOS, the number of PFOS molecules/cm^2^ is 70.6 and 9.6 for the untreated pipe section and BC 20% (70 °C), respectively. The number of PFOS molecules per unit surface area for a monolayer of coverage has been estimated to range from 4 to 20 molecules/nm², depending on whether the long axis of the molecule is parallel (4 molecules) or normal (20 molecules) to the surface.^77^ Assuming 20 PFOS molecules per cm^2^, for a monolayer of PFOS to be present indicates that on the untreated pipe section, PFOS molecules must be present in an arrangement beyond that of a monolayer. Furthermore, the maximum PFOS concentration removed from surfaces in the soaking experiment (Pipe I, BC20 (70 °C), 12 h, Figure S4) was measured at 35 μg/cm^2^, which corresponds to 421 PFOS molecules/nm^2^. This is yet another indicator of PFOS being stored in multiple layers in supramolecular assemblies. The differences between the number of molecules/nm^2^ estimated from the F concentration on the surfaces and the measurement in the soaking solution could be a result of the surface measurements being confined to 1500 TFU. The actual analytical depth goes beyond 1500 TFU; however, thereafter, hydrogen (H) measurements drop unrealistically, and C and oxygen (O) atoms overlap. This would lead to the elemental composition being skewed. The estimated number of F atoms/nm^2^, stored in a three-dimensional arrangement, is therefore likely to be higher than the 70 molecules/nm^2^ estimated above. Conversion of TFU into a metric scale is theoretically possible; however, it would lead to inaccuracies due to the unknown density of the AFFF layer. The ToF-ERD measurements do not provide information on the actual arrangement and/or orientation of molecules within these assemblies, and it is possible that molecules are not arranged perfectly in monolayers or bilayers but form tilted clusters.

### Environmental and Practical Implications

The results from the soaking experiment showed that PFAS removal from surfaces with heated BC (70 °C) was 2- to 8-fold more effective than TAP (70 °C) and 10- to 20-fold more effective than TAP at 20 °C based on single pipe sections. Thus, both the cleaning solution composition and temperature are crucial parameters for the decontamination of infrastructure from PFAS. These data correlate with the surface analysis using ToF-ERD, which revealed the most remaining F on the pipe surfaces after TAP treatments, and even the most effective treatment solution (BC20 (70 °C)) did not remove all PFAS from surfaces, despite the decreasing concentration of removed PFAS into solution during repetitive soaking intervals. The results align with other studies,^43,47^ highlighting the challenges of PFAS decontamination in fire suppression systems.

The static desorption experiment conducted herein does not necessarily reflect an actual decontamination scenario since flow-through setups are commonly used. Nguyen et al.^47^ compared flow-through conditions to lab-scale batch incubation experiments involving shaking and found that PFAS removal was slightly higher in flow-through experiments as compared to the batch tests. They furthermore showed that surface attrition reduces the PFAS rebound substantially. Thus, in full-scale decontamination, surface attrition (wherever applicable), e.g., pressure washing, in combination with heated BC solution might achieve even higher removal efficiency. An advantage of BC in aqueous solution, compared to other cleaning agents, is that residues of the cleaning solution will not negatively impact the system, since both BC and TAP are constituents of many AFFF and F3 foam products. Another advantage, compared to other solvents (e.g., MeOH or IPA), is that BC is associated with fewer hazards and precautionary statements under the GHS system,^78^ which is relevant for work safety. Decontamination costs are difficult to predict, since they depend on factors, such as the size of the fire suppression system, PFAS composition, and accessibility of the infrastructure, among others. However, decontamination is typically more cost-efficient and sustainable than replacing the infrastructure.

When evaluating PFAS concentrations with respect to AFFF contamination, target PFAS measurements, especially with a limited number of PFAS quantified, are insufficient, and techniques, such as the TOP assay and TF analyses, accounting for precursor PFAS, should be employed.^79−81^ The ToF-ERD data identified that the analysis of treatment solutions alone cannot provide evidence for successful decontamination of PFAS from fire suppression systems. Analysis of PFAS remaining on surfaces is required to determine whether decontamination has been successful to reflect the efficacy of treatment. Thus, measuring PFAS concentrations in solution does not reflect the mass of surface-bound PFAS remaining. Remaining PFAS on interior surfaces poses an ongoing risk of PFAS rebounding into F3 foams. PFAS rebound into F3 foams is expected to be greater than into TAP because many F3 foam products contain glycols in their formulations.^82−84^ Data describing the PFAS content of F3 foams following different decontamination approaches are scarce. The concentrations of PFAS in F3 foams are likely to rise over the period as the F3 foams are placed into fire suppression systems that held fluorinated foams as a result of slow rebound. Therefore, sampling these foams for PFAS immediately after they are placed in a fire suppression system would be of little value. Regulatory limits concerning PFAS levels in any firefighting foam^34,39,40^ could result in F3 foams eventually exceeding these levels as a result of insufficient decontamination, with 1.6 g/L of total PFAS detected in F3 foams one year after a double water rinsing.^41^

The combination of SEM and ToF-ERD data indicates that fluorinated supramolecular aggregates of PFAS exist on the interior pipe surfaces. These stable multilayered supramolecular forms of PFAS may account for the mass of PFAS calculated to be stored on the interior surfaces of the pipes. These multilayered structures represent a reservoir of PFAS that may delaminate over time and could account for the observed rebound effects. Regulators should take this into consideration when evaluating credible methods to prove decontamination.^85^ Ultimately, to determine treatment efficiency, it is essential to determine the total PFAS mass on pipe surfaces. Even though ToF-ERD is a valuable method to determine the remaining F on pipe surfaces, further efforts are necessary to facilitate and accelerate the surface analysis of PFAS mass.

## Supporting Information Available

Additional information on experimental setup, analytical methods (SEM, TOP assay, ToF-ERD), statistical analysis, and figures and tables complementing the analysis shown (PDF)
