# Correction to “Decontamination and Surface Analysis of PFAS-Contaminated Fire Suppression System Pipes: Effects of Cleaning Agents and Temperature”

**DOI:** 10.1021/acs.est.5c04388

**Published:** 2025-04-15

**Authors:** Björn Bonnet, Matthew K. Sharpe, Gulaim Seisenbaeva, Leo W. Y. Yeung, Ian Ross, Lutz Ahrens

The units reported in the section titled “Measurements of PFAS Supramolecular Assemblies” on page 2228, related to the number of molecules per unit surface area, should consistently be expressed as molecules/nm^2^ and nm^2^. However, some instances were incorrectly reported as molecules/cm^2^ and cm^2^. The reported unit μg/cm^2^ for the PFOS concentration (35 μg/cm^2^) measured for Pipe I, BC20 (70 °C) is correct. The corrected units are highlighted in bold as follows, starting from the bottom of the left text panel on page 2228.

The estimated number of F atoms/nm^2^ (see section S16) for the untreated AFFF-impacted surface is 1200, whereas the number of F **atoms/nm**^**2**^ for 20% BC (70 °C) is 163. Considering the AFFF layer mainly consists of PFOS, the number of PFOS **molecules/nm**^**2**^ is 70.6 and 9.6 for the untreated pipe section and BC 20% (70 °C), respectively. The number of PFOS molecules per unit surface area for a monolayer of coverage has been estimated to range from 4 to 20 molecules/nm^2^, depending on whether the long axis of the molecule is parallel (4 molecules) or normal (20 molecules) to the surface.^1^ Assuming 20 PFOS molecules per **nm**^**2**^, for a monolayer of PFOS to be present indicates that on the untreated pipe section, PFOS molecules must be present in an arrangement beyond that of a monolayer.

Author Leo W. Y. Yeung’s affiliation should read “MTM Research Centre, School of Science and Technology, Örebro University, Örebro 70182, Sweden.

No other correction was made to the paper.
