# Supplementary material for: Decontamination and Surface Analysis of PFAS-Contaminated Fire Suppression System Pipes: Effects of Cleaning Agents and Temperature
Source: Environ Sci Technol. 2025 Jan 23;59(4):2222–32. doi: 10.1021/acs.est.4c09474 (PMC11800388; doi:10.1021/acs.est.4c09474)
Supplement: Supplementary file 1 — es4c09474_si_001.pdf [file es4c09474_si_001.pdf]

Supplementary Information

Decontamination and surface analysis of PFAS-contaminated fire suppression system pipes: effects of cleaning agents and temperature

Björn Bonnet[a*], Matthew K. Sharpe[b], Gulaim Seisenbaeva[c], Leo W. Y. Yeung [d], Ian Ross[e] and Lutz Ahrens[a]

[a]Department of Aquatic Sciences and Assessment, Swedish University of Agricultural Sciences, 75651 Uppsala, Sweden

[b]Surrey Ion Beam Centre, University of Surrey, Guildford, Surrey, GU2 7XH, UK

[c]Department of Molecular Sciences, Swedish University of Agricultural Sciences, 75651 Uppsala, Sweden

[d]SMTM Research Centre, School of Science and Technology, Örebro University, 70182 Örebro, Sweden

[e]CDM Smith, 220 Montgomery Street. Suite 1418. San Francisco, CA 94104 USA, USA

*Email: bjorn.bonnet@slu.se

This document contains 31 pages, 18 figures and 11 tables.

S1: Pre-investigation of decommissioned stainless steel sprinkler system pipes

Two pre-investigations were undertaken to reduce the number of eligible pipes used for the experiment, with the goal of selecting the most contaminated pipes.

Pre-investigation 1: Ultrasonication supported methanol extraction.

Approximately 1 cm wide sections (diameter see Table S1) were cut off each pipe (*n* = 9). The cut outs were put into differently sized glass beakers (150-250 mL capacity) individually. Beakers were filled with methanol until the pipe section was completely covered (Table S1). Containers were placed into an ultrasonication bath for 1 hour. After sonication, methanol was analyzed for PFAS using 0.9 mL sample and 100 µL internal standard (IS) solution (for details see section 'PFAS analysis').

Table S1: Methanol extraction of stainless-steel pipe sections

ID/Label	Inner Diameter of pipe (cm)	Volume of methanol (mL)	Measured ∑PFAS (ng/mL)
A_MeOH	3.86	50	0.50
B_MeOH	3.86	50	0.40
D_MeOH	4.40	50	0.60
E_MeOH	3.00	40	168
F_MeOH	4.50	50	1760
G_MeOH	3.86	50	0.10
H_MeOH	3.85	50	1220
I_MeoH	5.70	70	1250
J_MeoH	3.00	40	36.8

The analysis showed that pipes F, H and I were the most PFAS-contaminated pipes and thus these pipe sections were selected for further experiments.

Pre-investigation 2: Scanning electron microscopy (SEM)

Approximately 1 cm x 1 cm pieces were cut out from pipes F, H and I for investigation using a scanning electron microscope (SEM). The goal behind this investigation was to visualize the structures of the AFFF-contaminated surfaces (Figure S1). Additionally, the outside of a stainless steel pipe was investigated as well. Multi-layered PFAS structures were previously identified on surfaces of sprinkler system pipes using SEM-EDX by Lang et al. (2022). Figure S1 showed that for all three pipes sections (F, H, I) the steel surface was completely covered compared to the the outside of a pipe section. SEM did not provide any precise resolution regarding the thickness of the layer, however, cracks in Figure S1 indicate a considerable depth of several. Figure S1 furthermore shows structures that could be associated with PFAS.

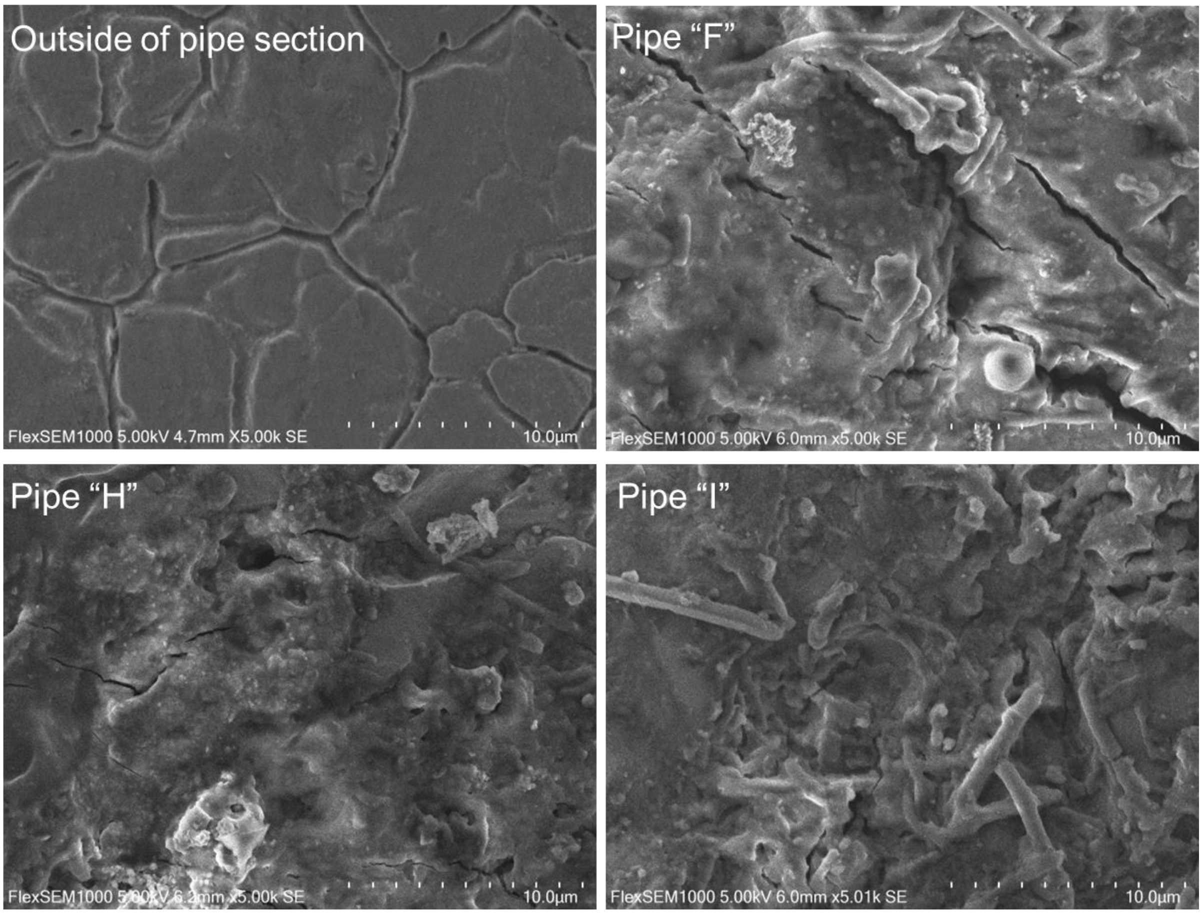

Figure S1: SEM images at 5000-fold magnification produced using Hitachi FlexSEM-1000 II microscope operating at 5 kV accelerating tension, working distance ca. 6 mm.

S2: Experimental Design – Soaking experiment

Multiple pipe sections (Table S2) were put into a PP-container to increase surface area. When placing the pipe sections into the container, it was ensured that the contaminated surface of each pipe section was always in full contact with the soaking solution and was not covered up by the other pipe sections. Pipe sections were completely covered within the soaking solution and volumes of soaking solutions were adjusted accordingly.

Table S2: Parameters of pipe sections and conditions for soaking experiment

Temperature [°C]	Cleaning solution	Pipe section	Number of pipe sections	Surface area at T1 [cm²]	Soaking Volume [mL]
20	MeOH	F	2	38.4	100
		H	4	64.3	150
		I	2	67.2	200
	TAP	F	2	39.0	100
		H	4	67.1	150
		I	2	68.0	200
	BC10	F	2	37.8	100
		H	4	66.2	150
		I	2	66.4	200
	BC20	F	2	38.4	100
		H	4	68.3	150
		I	2	67.2	200
40	TAP	F	2	39.6	100
		H	4	67.5	150
		I	2	64.8	200
	BC10	F	2	38.7	100
		H	4	62.8	150
		I	2	67.2	200
	BC20	F	2	37.8	100
		H	4	66.1	150
		I	2	65.6	200
70	TAP	F	2	38.4	100
		H	4	65.9	150
		I	2	67.2	200
	BC10	F	2	38.4	100
		H	4	66.5	150
		I	2	66.4	200
	BC20	F	2	38.9	100
		H	4	64.7	150
		I	2	68.1	200

The soaking experiment was performed at three different temperatures: Room temperature (20°C), 40°C and 70°C. Experiments at room temperature were performed under a fume hood at laboratory conditions and elevated temperatures were conducted within temperature-controlled ovens (40°C: Nabertherm, N-54E; 70°C: VWR, Dry-Line 56 Prime) set to the respective temperature. Temperature was measured throughout the whole experimental runtime.

Pipe sections of pipe H were used for a follow-up experiment using time-of-flight elastic recoil detection (ToF-ERD) (see section 'S8 Surface analysis with ToF-ERD'). To enable a surface analysis of each time point without interrupting the soaking experiment, it was necessary to remove one pipe section of pipe H from the container after each time point. Therefore, the total surface area interacting with the soaking solution was reduced by the area of the removed pipe section. Calculated concentrations were adjusted for this change in surface area.

S3: PFAS target compounds and target analysis

A total of 24 PFAS were analyzed including 11 perfluoroalkyl carboxylic acids (PFCAs) (perfluorobutanoic acid (PFBA), perfluoropentanoic acid (PFPeA), perfluorohexanoic acid (PFHxA), perfluorohepanoic acid (PFHpA), perfluorooctanoic acid (PFOA), perfluorononanoic acid (PFNA), perfluorodecanoic acid (PFDA), perfluoroundecanoic acid (PFUnDA), perfluorododecanoic acid (PFDoDA), perfluorotridecanoic acid (PFTriDa) and perfluorotetradecanoic acid (PFTeDA)), 7 perfluorosulfonic acids (PFSAs) (perfluorobutanesulfonic acid PFBS, perfluoropentanesulfonic acid (PFPeS), perfluorohexanesulfonic acid (PFHxS), perfluorohepanesulfonic acid (PFHpS), perfluorooctanesulfonic acid (PFOS), perfluorononanesulfonic acid (PFNS) and perfluorodecanesulfonic acid (PFDS)), 3 fluorotelomer sulfonates (4:2 FTSA, 6:2 FTSA and 8:2 FTSA), N-methyl-perfluorooctane sulfonamido acetic acid (Me-FOSAA), N-ethyl-perfluorooctane sulfonamido acetic acid (Et-FOSAA) and perfluorooctane sulfonamide (FOSA).

All samples from the soaking experiments were prepared for direct injection analysis by ultraperformance liquid chromatography coupled to tandem mass-spectrometry (UPLC-MS/MS) analysis (Sciex Triple Quad™ 3500 LC-MS/MS, USA) (for details see Smith et al., 2022). For aqueous samples (TAP, BC10, BC20), 250 µL sample, 250 µL MilliQ, 400 µL methanol and 100 µL IS-solution in methanol were used, while for methanol-based samples, 250 µL sample, 150 µL methanol, 500 µL MilliQ and 100 µL IS-solution in methanol were added to a 1.7 mL PP autoinjector vial and vortexed. Due to exceeding the linear range of the calibration curve for PFOA, PFHxS and PFOS in for some samples, a diluted series with 25 µL sample was prepared.

Table S3: Limits of detection (LOD) and limits of quantification (LOQ) in (ng/mL)

	TAP		BC10		BC20		MeOH	
	LOD	LOQ	LOD	LOQ	LOD	LOQ	LOD	LOQ
PFBA	0.01	0.03	0.01	0.03	0.01	0.03	0.01	0.03
PFPeA	0.01	0.03	0.01	0.03	0.01	0.03	0.01	0.03
PFHxA	0.01	0.03	0.01	0.03	0.01	0.03	0.01	0.03
PFHpA	0.01	0.03	0.01	0.03	0.01	0.03	0.01	0.03
PFOA	0.01	0.03	0.01	0.03	0.01	0.03	0.01	0.03
PFNA	0.25	0.72	0.01	0.03	0.22	0.63	0.01	0.03
PFDA	0.01	0.03	0.22	0.57	0.04	0.10	0.67	1.95
PFUnDA	0.05	0.12	0.01	0.03	0.18	0.52	0.18	0.45
PFDoDA	0.10	0.25	0.08	0.22	0.07	0.19	0.12	0.34
PFTriDA	0.01	0.03	0.01	0.03	0.01	0.03	0.01	0.03
PFTeDA	0.06	0.15	0.01	0.03	0.01	0.03	0.08	0.23
PFBS	0.03	0.08	0.06	0.15	0.02	0.03	0.07	0.15
PFPeS	0.07	0.18	0.02	0.04	0.04	0.08	0.14	0.36
PFHxS	0.02	0.05	0.01	0.03	0.01	0.03	0.12	0.33
PFHpS	0.10	0.28	0.01	0.03	0.01	0.03	0.11	0.31
PFOS	0.05	0.14	0.02	0.03	0.03	0.07	0.39	1.07
PFNS	0.06	0.14	0.01	0.02	0.04	0.09	0.08	0.20
PFDS	0.07	0.16	0.03	0.05	0.07	0.17	0.09	0.15
4:2 FTSA	0.01	0.03	0.01	0.03	0.01	0.03	0.01	0.03
6:2 FTSA	0.24	0.68	0.33	0.75	0.43	1.19	0.12	0.17
8:2 FTSA	0.01	0.03	0.01	0.03	0.01	0.03	0.16	0.46
FOSA	0.04	0.10	0.01	0.03	0.01	0.03	0.04	0.09
Me-FOSAA	0.01	0.03	0.01	0.03	0.01	0.03	0.07	0.19
Et-FOSAA	0.04	0.10	0.01	0.03	0.05	0.14	0.19	0.55

Table S4: Method relative recoveries in (%)

	Soaking experiment	TOP assay		Soaking experiment	TOP assay
PFBA	77%	95%	PFOS	103%	89%
PFPeA	145%	90%	PFNS	103%	89%
PFHxA	78%	88%	PFDS	103%	89%
PFHpA	80%	85%	4-2FTSA	86%	90%
PFOA	81%	93%	6-2 FTSA	74%	87%
PFNA	98%	89%	8-2 FTSA	70%	79%
PFDA	73%	83%	FOSA	73%	88%
PFUnDA	71%	86%	Me-FOSAA	85%	84%
PFDoDA	78%	90%	Et-FOSAA	75%	85%
PFTriDA	74%	95%			
PFTeDA	74%	95%			
PFBS	77%	88%			
PFPeS	82%	85%			
PFHxS	82%	85%			
PFHpS	103%	89%			

S4: Total oxidizable precursor assay – spiking test

A total oxidizable precursor (TOP) assay was performed to estimate contribution of precursor PFAS not identified in the targeted analysis (for details see Houtz&Sedlak, 2012). Due to the addition of BC, an organic compound, interferences during oxidation in the TOP assay inhibiting oxidation of PFAS precursors were expected. Hence, a spiking experiment with a known concentration of an oxidizable PFAS precursor (6:2 FTSA) was performed in MilliQ water and in an aqueous solution containing 20% BC as in the soaking experiment. 100 µL of 6:2 FTSA in methanol (c = 32 µg/mL) was spiked into a 15 mL PP-tube and brought to dryness under N-stream. One set of duplicates was reconstituted in 100 µL MQ-water and another set in 100 µL MilliQ-water containing 20% BC. TOP assay was performed for three different dosages of oxidant potassium persulphate ($K_2O_8S_2$) and base sodium hydroxide solution (NaOH) (Table S5). The lowest dosage (60 mM $K_2O_8S_2$/150mM NaOH) used according to the TOP assay originally proposed by Houtz&Sedlak (2012). Additionally, dosages of 120 mM $K_2O_8S_2$/300 mM NaOH and 180 mM $K_2O_8S_2$/450 mM NaOH were prepared. Oxidant and base were added from prepared stock solution of 200 mM $K_2O_8S_2$ and 9 M NaOH, MQ water was added to a final volume of 2 mL. Blanks were prepared consisting of 100 µL MQ water instead of sample. Control samples of 6:2 FTSA without oxidant and base in only MilliQ-water were prepared in triplicates. All samples were put into a temperature-controlled water bath at 85 °C and removed after 6 hours. After reaching room temperature, pH was estimated using indicator paper (ROTA®, pH 1-14, VWR, Belgium), 50 µL of methanol and different volumes of 6 M HCL were added to obtain a target pH between 4-7 (Table S5).

Table S5: TOP Assay – spiking test

200 mM $K_2O_8S_2$ (µL)	9 mM NaOH (µL)	Sample (µL)	MilliQ (µL)	Final Volume (µL)
600	34	100	1266	2000
1200	67	100	633	2000
1800	100	100	-	2000

The spiking test with 6:2 FTSA in MilliQ water and MilliQ water containing 20% BC (BC20) was performed to test if oxidation of 6:2 FTSA will be inhibited due to the presence of BC. The tests indicate that oxidation was not only inhibited but completely prevented if BC was present in the solution (Table S6). The concentrations of the spiked substance 6:2 FTSA as well as their predominantly found product PFCAs after oxidation during the TOP assay for three different dosages (i.e. 60/150, 120/300 and 180/450) of oxidant ($K_2S_2O_8$) and base (NaOH) are presented in Table S6. For spiking tests in MilliQ water, initially spiked 6:2 FTSA concentration (32000 ng/mL) was reduced to an average concentration of 458 ng/mL, 148 ng/mL and 161 ng/mL for 60/150, 120/300 and 180/450 ($K_2S_2O_8$/NaOH), respectively. For BC20, concentrations of initially spiked 6:2 FTSA remained at the initially spiked level and did not produce any PFCA product above the LOD. Increasing dosages of oxidant and base did not have any effect on successful oxidation during the TOP assay either. We conclude from these spiking tests that PFAS present in samples from the soaking experiment including BC are not going to be successfully oxidized during the TOP assay. Therefore, we decided to include only TAP samples and MeOH samples for TOP assays. To account for contribution of precursors in samples containing BC, we decided to determine total fluorine (TF) content by combustion-ion-chromatography (CIC).

Table S6: Concentration of spiked 6:2 FTSA and its products after oxidation in TOP assay

	60/150		120/300		180/450	
ng/mL	BC20	MilliQ	BC20	MilliQ	BC20	MilliQ
6:2-FTSA	31655	458	31456	148	31834	161
PFBA	<LOD	3136	<LOD	3002	<LOD	2829
PFPeA	<LOD	5486	<LOD	4887	<LOD	4717
PFHxA	<LOD	3884	<LOD	3797	<LOD	3801
PFHpA	<LOD	627	<LOD	695	<LOD	594

Table S7: pH and neutralization after oxidation for TOP assay spiking test with 6M HCL

	60/150				120/300				180/450			
Replicate	1		2		1		2		1		2	
	Vol.HCL (µL)	pH	Vol. HCL (µL)	pH	Vol. HCL (µL)	pH	Vol. HCL (µL)	pH	Vol. HCL (µL)	pH	Vol. HCL (µL)	pH
MilliQ before neutralization	0	14	0	14	0	14	0	14	0	14	-	14
MilliQ after neutralization	10	6-7	10	6-7	25	6-7	20	6-7	30	6	30	6
BC20 before neutralization	0	14	0	14	0	14	0	14	0	14	0	14
BC20 after neutralization	5	6-7	5	6-7	10	7	10	7	10	6	10	6

S5: Total oxidizable precursor assay – real samples

Table S8: TOP assay on 12 h samples – volumes and concentrations of TOP assay constituents

200 mM K2O8S2 (µL)	9 mM NaOH (µL)	Sample (µL)	MilliQ (µL)	Final Volume (µL)
600	34	1000	366	2000

Table S9: pH and neutralization of TOP assay for T1 samples with 6M HCL

Sample ID	F		H		I	
Replicate	1	2	1	2	1	2
	Vol. HCL (µL)	pH	Vol. HCL (µL)	pH	Vol. HCL (µL)	pH
TAP before neutralization	0	14	0	14	0	14
TAP after neutralization	10	7	10	7	10	6-7
TAP40 before neutralization	0	14	0	14	0	14
TAP40 after neutralization	10	7	10	7	10	7
TAP70 before neutralization	0	14	0	14	0	14
TAP70 after neutralization	10	7	10	7	10	7
MeOH before neutralization	0	14	0	14	0	14
MeOH after neutralization	10	2	5	6	5	7
Blank before neutralization	0	14	0	14	0	14
Blank after neutralization	5	7	10	7	10	7

S6: Total fluorine (TF) analysis

The system is consisted of a combustion module (Analytikjena, Germany), a 920 absorbent module, and a 930 Compact IC flex (Metrohm, Switzerland). The separation of anions was performed on an ion-exchange column (Metrosep A Supp5, 4 mm x 150 mm) with isocratic elution using the following eluent (64 mM sodium carbonate and 20 mM sodium bicarbonate). In brief, 100 µL of the liquid samples was placed on a quartz glass boat and combusted with hydropyrolysis at 1050 °C. During combustion, all fluorine was converted into hydrogen fluoride (HF) and were absorbed into ultrapure water (18.2 MΩ). The levels of fluoride ions in the solution were analyzed using the ion chromatograph with conductivity detector. Since background fluoride contamination was noted in daily measurement, the measurement of TF started until low variation (RSD < 10%) of background combustion blanks (empty quartz glass boat) were obtained. The TF results were obtained using a five-point external calibration curve (50 to 1000 ng/mL F) prepared from solid PFOA potassium salt (Fluka, Hampton, United States). Quantification of fluoride was performed by subtracting the peak area of fluoride in the combustion blanks between samples and then using a five-point external calibration curve for calculation. The peak areas of the calibration curve had been also subtracted from that of the combustion blank. Quality control standard using 250 ng F/mL PFOA standard was used in between 10 samples to evaluate the stability of the instrument; the reported concentrations of the QC standard was 236 ng F/mL with the relative standard deviation of 24%. Blank samples of TAP, BC10 and BC20 were evaluated and subtracted, and their levels were found to be 1.4, 0,63 and 0,53 ng F/L, respectively.

S7: Data conversion for F-equivalent

Since CIC only measures the F content (both organic bound or inorganic fluoride) of a sample, to compare the results from target PFAS and TOP assay analyses, concentrations of PFAS have to be converted into F-equivalent concentration using the following formula:

$$c_F = \frac{n_F MW_F}{MW_{PFAS}} \times c_{PFAS}$$

where C_F is the corresponding fluoride level (ng/mL F), n_F stands for the numbers of fluorine in the PFAS, MW_F is the molecular weight of fluorine, MW_{PFAS} stands for the molecular weight of PFAS and C_{PFAS} is the quantifiable PFAS concentration in target PFAS or TOP assay analyses.

S8: Surface analysis with ToF-ERD:

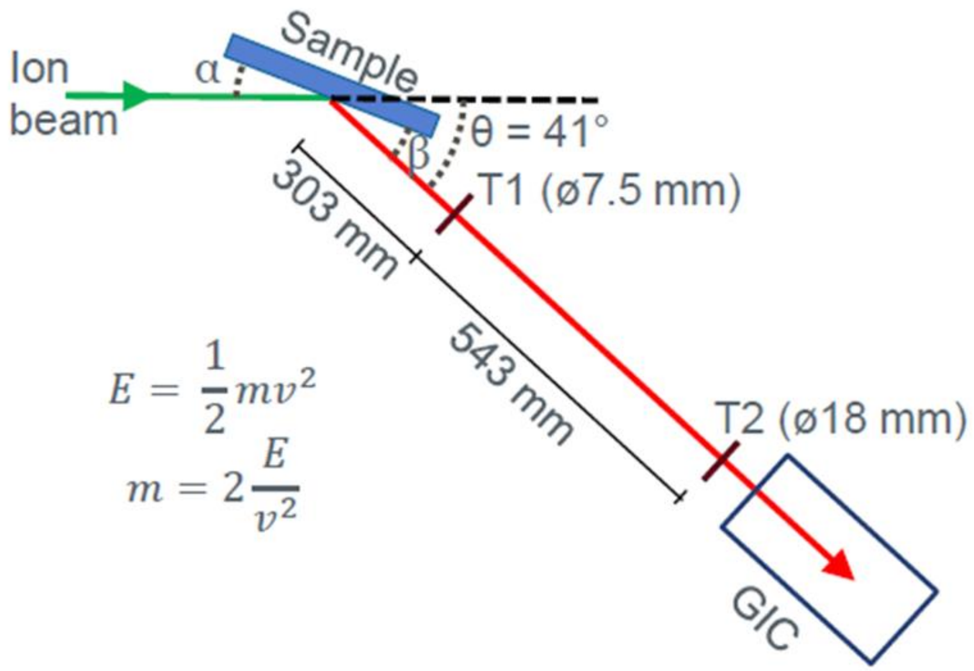

Figure S2: Schematic of ToF-ERD setup. Incident angle (α) and exit angle (β) equal to 69.5° from sample normal. Timing gates, T1 and T2, used for detecting time-of-flight of recoiled atoms, with Gas Ionisation Chamber (GIC) used for detecting energy of recoiled atoms.

Figure S2 in SI illustrates the time-of-flight elastic recoil detection (ToF-ERD) configuration at the Ion Beam Center at Surrey University[1]. This setup features two timing foils for time-of-flight measurements and a gas ionization chamber (GIC) for assessing energy. For this study, the scattering angle was set at 41°, with the incident and exit angle at 69.5° to maximize sensitivity, mass, and depth resolution throughout the whole sample[2]. A HVE 860 negative sputter ion source generates negatively charged ions that an injector magnet then leads into a 2 MV tandem accelerator, accelerating the ions to a designated energy and converting them to a positive charge. The experiment utilized 16 MeV $^{127}I^{8+}$ ion beams with an energy dispersion under 10 keV, chosen for optimal cross-sections, count rate, and mass differentiation. The ToF-ERD experiment ran for 20 min for each sample with a 3 x 4 mm² beam spot, adjustable by slits prior to entering the sample chamber. Coincidence counts between the second timing foil and the GIC were approximately 900 counts/s, against a background of less than 20 counts/s. The ToF-ERD histograms were processed with Potku software (Build 2.2.4)[3] to remove any spurious coincidences caused by incorrect ToF readings, producing elemental depth profiles. Pristine stainless steel which never was in contact with AFFF was analyzed as a blank.

S9: Results Soaking experiment

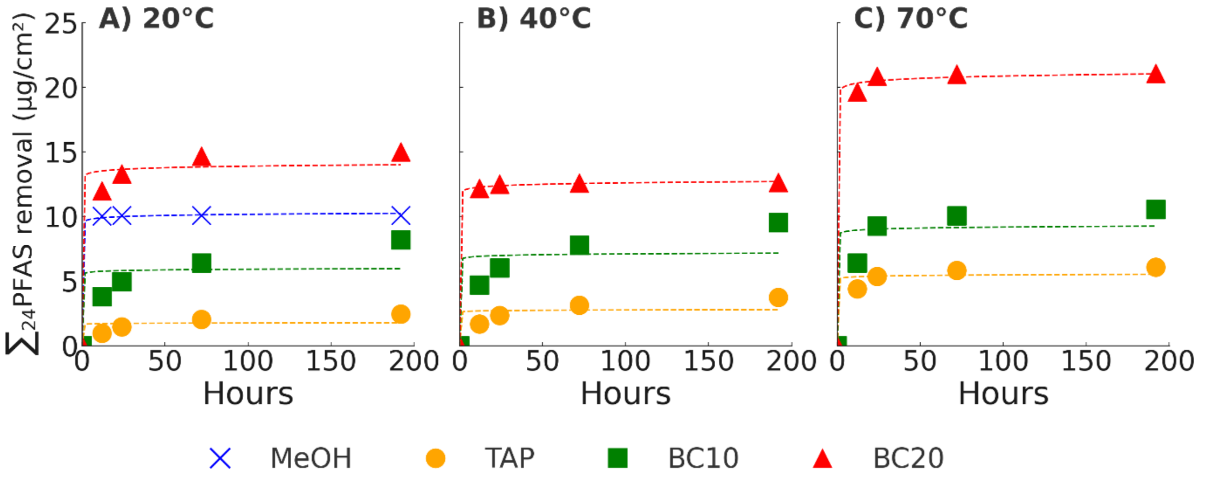

Figure S3: Accumulated PFAS removal (μg/cm²) from stainless steel pipes using methanol (MeOH) (only 20°C), tap water (TAP), 10 wt% butyl carbitol in TAP (BC10) and 20 wt% BC in TAP (BC20), respectively, during soaking experiments at A) 20°C, B) 40°C and C) 70°C. Data points represent average concentrations (n=3). Fitted logarithmic functions are presented in table S11.

Table S10: Fitted logarithmic functions and R^2 values for soaking experiment

Treatment	Temperature	Equation	R^2
MeOH	20	343.3ln(x)+8745.19	0.9941
TAP	20	62.35ln(x)+1508.09	0.7297
BC10	20	209ln(x)+5088.93	0.7873
BC20	20	475.68ln(x)+11953.36	0.9863
TAP	40	97.65ln(x)+2376.4	0.7823
BC10	40	250.19ln(x)+6112.13	0.8155
BC20	40	426.37ln(x)+10842	0.9970
TAP	70	188.33ln(x)+4712.9	0.9668
BC10	70	318.14ln(x)+7899.16	0.9076
BC20	70	707.26ln(x)+17954.01	0.9977

S10: Trend for chain length and head group dependent desorption of PFAS

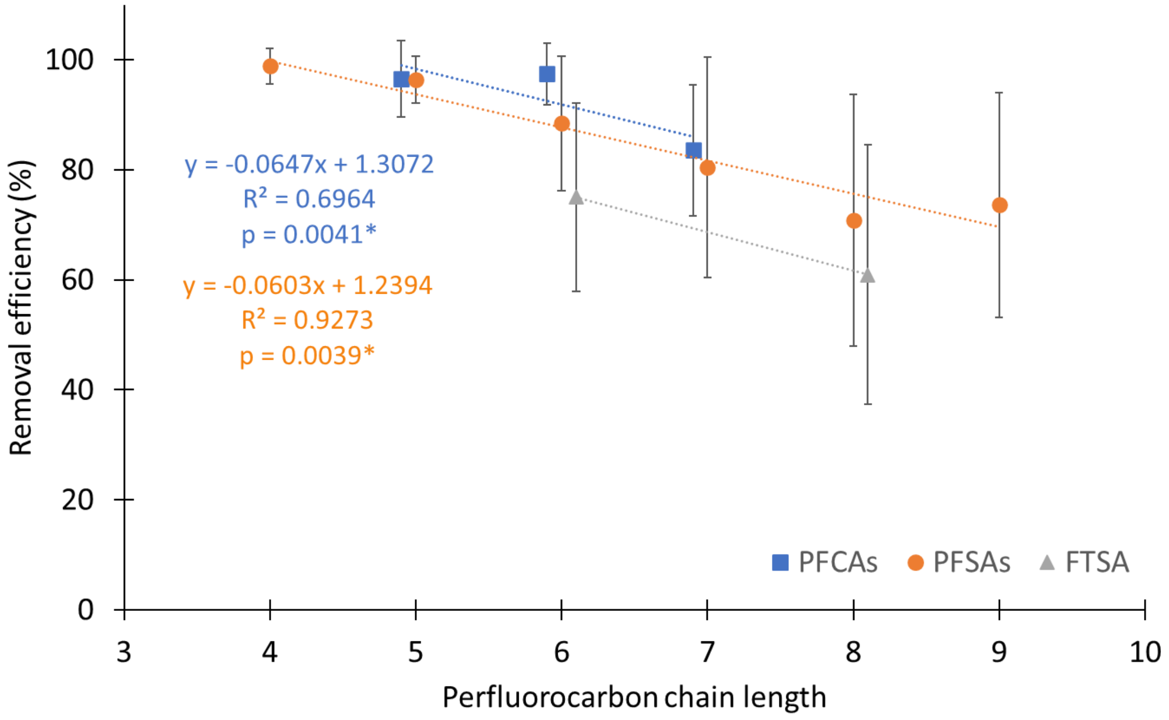

Figure S4: Removal efficiency (%) (∑PFAS 12h/∑PFAS 192h * 100) as a function of perfluorocarbon chain length. Data points represent subgroup averages of values across all treatments. Only compounds with 100% detection frequency (n = 10 per data point) are presented. *p-values are calculated based upon all available data points.

The removal of PFAS was dependent on the perfluorocarbon chain length and functional group comparing TAP, BC10, BC20 and MeOH after 192 h (Figure S4 in SI). After 192 h, the average removal decreased by 6.6 ± 7.6% for each CF_2 moiety for C_5-C_7 PFCAs and 5.6 ± 2.9% for C_4-C_9 PFSA. This indicates that shorter chain compounds are released into solution more easily than longer chain compounds under the same experimental conditions. Krafft Point (T_K) is the temperature at which the solubility of a surfactant is equal to the critical micelle concentration (CMC)[4, 5]. T_K increases with increasing number of carbons in alkyl chain[4]. In addition, each additional CH_2 moiety increases the hydrophobicity and surface activity of the surfactant[4] and thus longer chain PFAS show a stronger interaction with surfaces. When comparing the functional groups, we generally observed the trend with increasing removal efficiency for PFCAs > PFSAs > FTSA. The removal efficiency was 4.7 ± 2.9% higher for PFCAs compared to PFSAs and 17 ± 0.87% higher for PFSAs compared to FTSAs. Removal efficiency was 22% higher for PFHxA than for 6:2 FTSA and 10% higher for PFOS than for 8:2 FTSA Dahlblom et al. found decreasing PFAS removal for compounds with increasing chain length from contaminated fire suppression materials, however, the assessment of removal

efficiency differed between the present study. This is in accordance with previous studies which investigated the (de)sorption kinetics of PFAS on soils[6, 7].

S11: Observations on single pipe sections and rebound experiment

Figure S5 presents the PFAS concentration in solution expressed in terms of surface area of pipe that they evolved from for all individual pipe sections (F, H and I; not averaged). The four fading green bars indicate the different soaking intervals (12 h, 24 h, 72 h and 192 h). We observed the highest PFAS removal into solution within the first soaking interval (12 h) and generally saw a decreasing removal in the following time intervals. We furthermore observed that the amount of PFAS removed from surfaces differed between the individual pipe sections. The PFAS removal from pipe section I was generally higher than for pipe sections F and H. The differences became most rigorous in the BC20 scenarios. The three maximum concentrations removed into solution in the entire soaking experiment were observed for pipe I in BC20 (20°C), BC20 (40°C) and BC20 (70°C) with 20400 ng/cm², 21800 ng/cm² an 40000 ng/cm² respectively. The factor by which the total removed PFAS increased between pipe F and H to pipe I were 2-3 (BC20 (20°C) and BC20 (40°C)) and 4-5 in BC20 (70°C). Reasons for why there were more PFAS removed from pipe I could be either that pipe I was more contaminated in the first place, or that the experimental conditions were more effective for pipe I, in the sense that the conditions were more closely to the overall T_K of PFAS assemblies on pipe I.

The red bars in Figure S5 represent the PFAS removed from surfaces during the rebound experiment (rebound experiment was not performed for pipe F). The highest concentrations were found for pipe I previously soaked in BC10 (20°C), TAP (20°C) and TAP (40°C) with 1990 ng/cm², 1350 ng/cm² and 1077 ng/cm² respectively. We observe that PFAS rebound was generally higher for pipe sections which received a less efficient treatment in the first place. For pipe I treated with TAP (20°C), PFAS rebound was almost as high as the initial soak (1490 ng/cm²). PFAS rebound was less pronounced for treatments at 70°C and with BC20 (all temperatures). The rebound experiment was only performed in TAP and only for 7 days. In the future it would be necessary to perform rebound experiment over a longer period (Dahlbom et al. 2024) and into actual F3 foam to represent a more realistic scenario. However, we can conclude from our soaking experiments that PFAS dissolved more efficiently into solutions containing BC. Since BC is a major constituent of F3 foams, we expect higher rebound into F3 foams. Considering long residence time within the system, PFAS rebound might happen over several years.

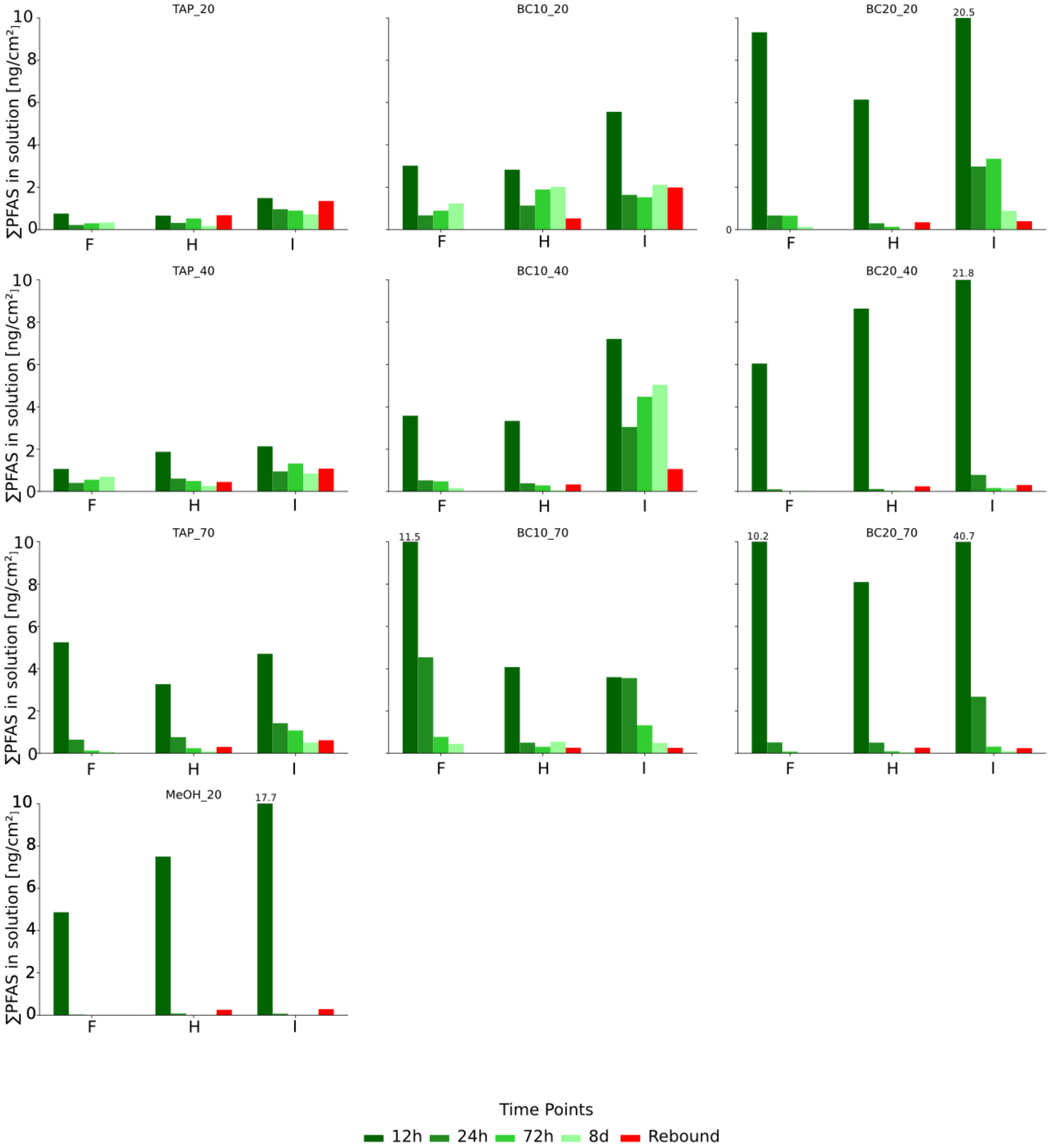

Figure S5: PFAS concentrations from soaking experiment expressed for single pipe sections including rebound test. Rebound test concentrations were measured after 1 week of incubation in tap water.

S12: TOP assay:

Comparison of PFAS concentration before and after TOP assay:

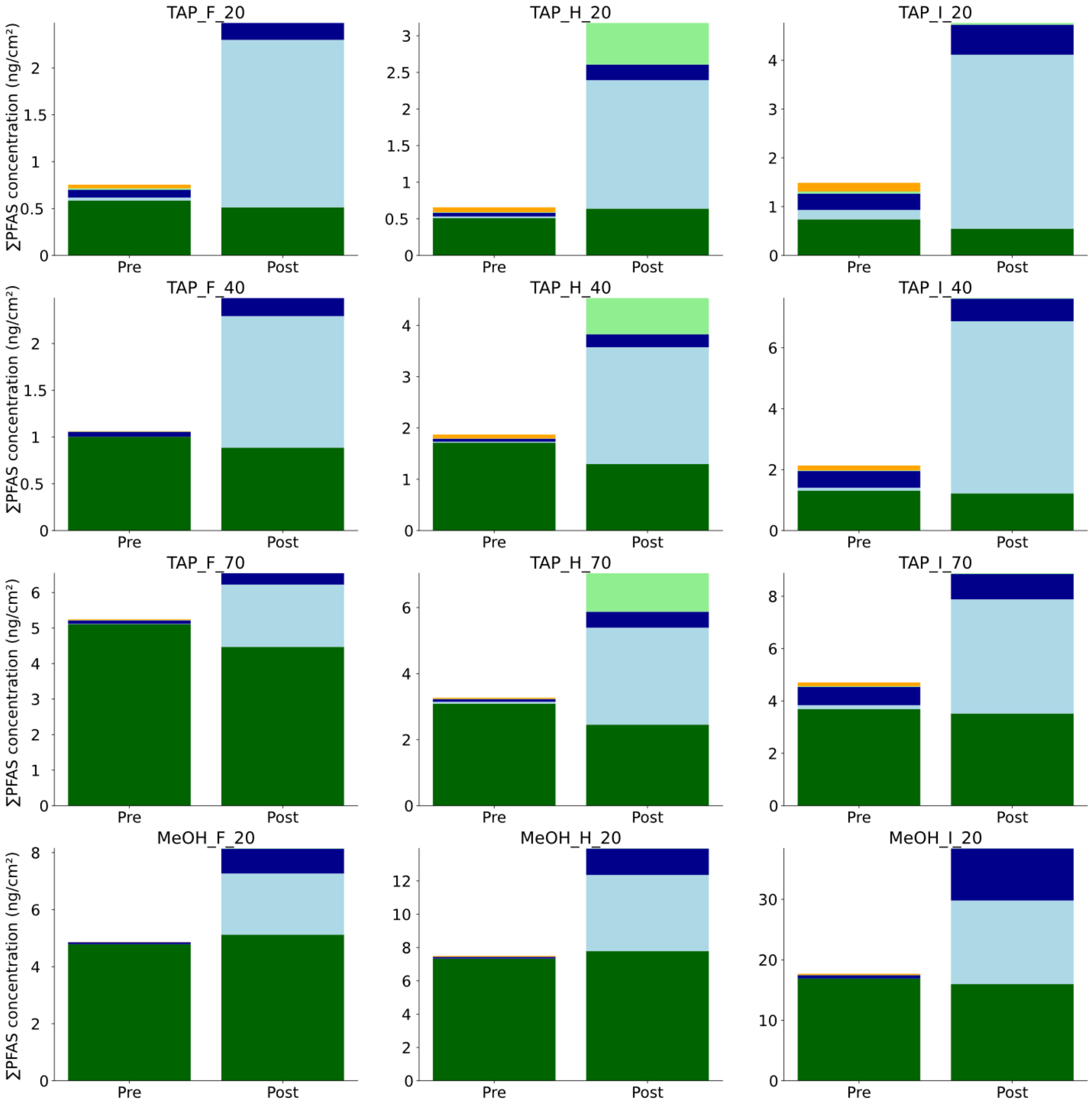

Figure S6: Comparison of ∑PFAS concentration before (pre) and after (post) oxidation in TOP assay. Y-axis in ng/cm^2.

For targeted analysis, the overall dominant fraction of PFAS were long chain PFSAs (i.e. PFOS, PFHxS, PFHpS), which contributed 68% (TAP (20°C)) to 97% (MeOH (20°C)) of total PFAS (Figure S6 in SI). After oxidation the ratio shifted and overall contribution of PFSAs decreased to 17% (TAP (20°C)) to 53% (MeOH (20°C)). Short chain PFSAs (i.e. PFBS, PFPeS) in targeted analysis contributed to a maximum of 2% in TAP (20°C). After oxidation, contribution of short chain PFSAs increased to a maximum contribution of 6.2% (TAP (20°C)). The highest contribution of short chain PFCAs accounted for 6.8% (TAP (20°C)) in targeted analysis and increased to an overall contribution of 32% (MeOH (20°C)) up to 67% (TAP

(20°C)) using TOP assay. Long chain PFCAs ranged between 2.1% (MeOH (20°C)) and 14% (TAP (20°C)) in targeted analysis and ranged between 6.1% (TAP (40°C)) and 15% (MeOH (20°C)) after oxidation. Other PFAS (i.e. detected 6:2 and 8:2 FTSA) contributed between 0.68% and 9.1% in targeted analysis and were not detected after oxidation which is expected since PFAS precursors have shown to break down during TOP assay[8].

S13: ToF-ERD measurements:

General information of histograms and depth profiles

Histograms and depth profiles of ToF-ERD measurements for all samples are presented in Figures S7 to S16. The histograms display both the composition as well as the intensity (counts/s) (referring to the color scale) of elements detected on the surface of each sample. Elements represented in the plot are the measurements of velocity (x-axis) and energy (y-axis). The curved shape of the plot for each element results from differences in velocity and energy for detected atoms (relating to the atoms mass from the kinetic energy $E = \frac{1}{2}mv^2$). A detection at the tip (top left) of the curve indicates a measurement with both high velocity and energy, whereas atoms detected towards the bottom of the curve (bottom right) are lower in both velocity and energy. Differences in velocity and energy for the same element result from interferences with the electron clouds of other elements after they have been recoiled by the ion beam. Interferences with electron clouds of other elements are less pronounced for atoms sitting at the very surface of the sample compared to atoms further in. Hence, we obtained depth position information of the atom within the sample. This information is further specified within the depth profiles. Depth profiles display the elemental concentration (y-axis) at a particular depth within the sample (x-axis).

Remaining F, C and Fe during soaking experiment

At 20°C remaining F concentration after 192 h were measured with 2.6 at.%, 4.7 at.%, 2.2 at.% and 2.1 at.% for TAP (20°C), BC10 (20°C), MeOH (20°C) and BC20 (20°C). At 40°C remaining F on pipe surfaces was highest for BC20 (40°) with 2.3 at.%, whereas TAP (40°C) and BC10 (40°C) showed 1.9 at.% remaining F after 192 h of treatment (Table S12).

For C, we generally observed a decrease between the initial concentration (untreated) and the concentration after 8 days of treatment. The three highest remaining C concentration on the pipe sections after treatment were observed for TAP (40°C), TAP (20°C) and BC (20°C) with 28% (50 counts/s), 27% (50 counts/s) and 26% (50 counts/s) respectively. The lowest C concentrations remaining on pipe surfaces besides treatment BC20 (70°C) were observed for MeOH (20°C) with 19% (30 counts/s) and BC10 (70°C) with 18% (30 counts/s).

For Fe, we generally observed an increase between the initial (untreated) concentration and the concentration after 8 days of treatment. Highest Fe concentration after 8 days of treatment were measured for BC20 (20°C) with 35% (50 counts/s), MeOH_20 with 28% (30 counts/s) and BC10 (70°C) with 25.9% (30 counts/s). The lowest Fe concentration after 8 days of treatment were measured for TAP (40°C), TAP (20°C) and BC10 (20°C) with 9.4% (10 counts/s), 12% (10 counts/s) and 16% (20 counts/s) respectively.

For TAP treatment at all temperatures as well as for BC10 (20°C), BC10 (40°C) and BC20 (40°C), we observed constant detection of all elements throughout the entire analytical depth, especially for Fe we did not observe a significant increase in detection, indicating it is covered up by the AFFF associated layer.

For MeOH (20°C), we observed concentration of Fe starting to increase and of C starting to decrease at a depth of around 500 TFU and 250 TFU after 24 h and 192 h of treatment respectively. F was detected constantly.

For BC10 (70°C), Fe concentration was starting to increase after 72 h of treatment at a depth of around 1000 TFU and yielding 25% at a depth of 1500 TFU. After 192 h of treatment, Fe started to increase at 750 TFU and reaches 30% at 1500 TFU. C concentrations began to decrease at the same depth, whereas F concentrations are measured constantly throughout all treatments with decreasing intensity after 192 h of treatment.

In BC20 treatments, we observed an increasing Fe and decreasing C concentration for BC20 (20°C) starting after 24 h of treatment at a depth of about 750 TFU and even earlier in after 192 h. F was measured constantly throughout the entire analytical depth with lower intensity after 192h of treatment. For BC20 (70°C) this trend was most distinct. Fe started to increase at a depth between 100 TFU to 250 TFU after 24 h of treatment and thereafter. For BC20 (70°C) after 192 h of treatment the Fe increased is most significant and reaches an elemental concentration of 45% at a depth of 1500 TFU. C showed decreasing concentration at similar analytical depths and F concentration decreases with increasing treatment time.

Discussion about the extent of the analyzed surface area

Five measurements of untreated pipe sections by ToF-ERD showed that the initial concentration of F, C and Fe may varied between 3.4 at.% - 8.0 at.%, 26 at.% - 32 at.% and 5.4 at.% - 21 at.% respectively, confirming variation of elemental concentration. Furthermore, ToF-ERD measurements throughout the treatments were performed on a ca. 10 mm x 10 mm piece cut off from a larger subsection (ca. 3 cm x 6 cm) of the initial pipe (see Figure S7 in SI). Therefore, pieces analyzed by ToF-ERD did have several cm between them. The actual area analyzed by ToF-ERD is approximately 3 mm x 4 mm (Figure S8 in SI). Despite the high

accuracy of ToF-ERD measurements themselves, the fact that only a 3 mm x 4 mm area is analyzed will lead to higher measurement uncertainties with respect to the soaking experiment, where there are substantially larger surface areas interacting with the soaking solution. It could have been more representative to cut out 10 mm x 10 mm pieces right next to each other. This would, however, have been less practical during the soaking experiment. It is recommended to take these factors into account for future analysis

Table S11: Elemental concentration of F, C and Fe on pipe sections measured by ToF-ERD presented in atomic % during the time points T1 (12 h), T2 (24 h), T3 (72 h) and T4 (192 h)

<table>
<tr><th colspan="3">pristine steel (blank)</th><th colspan="3">Untreated (T0)</th><th rowspan="2"></th><th colspan="3">MeOH_20</th></tr>
<tr><td>F</td><td>C</td><td>Fe</td><td>F</td><td>C</td><td>Fe</td><td>F</td><td>C</td><td>Fe</td></tr>
<tr><td rowspan="4">0.1</td><td rowspan="4">9.3</td><td rowspan="4">52.4</td><td rowspan="4">5.7 ± 2.3</td><td rowspan="4">28.7 ± 2.9</td><td rowspan="4">13.1 ± 7.7</td><td>T1</td><td>2.5</td><td>23.2</td><td>9.8</td></tr>
<tr><td>T2</td><td>1.5</td><td>18.8</td><td>25.4</td></tr>
<tr><td>T3</td><td>2.2</td><td>20.5</td><td>19.9</td></tr>
<tr><td>T4</td><td>2.2</td><td>18.5</td><td>28.3</td></tr>
</table>

	**TAP_20**			**BC10_20**			**BC20_20**		
	F	C	Fe	F	C	Fe	F	C	Fe
T1	4.5	25.5	13.6	7.7	27.8	9.0	3.5	25.8	13.6
T2	2.3	25.1	12.1	7.3	29.7	7.2	2.2	19.5	34.0
T3	1.5	24.1	14.9	5.0	30.4	7.0	1.8	18.1	25.6
T4	2.6	26.9	12.0	4.7	26.1	15.8	2.1	19.7	35.4
	**TAP_40**			**BC10_40**			**BC20_40**		
	F	C	Fe	F	C	Fe	F	C	Fe
T1	2.5	22.7	18.9	1.7	19.9	26.0	1.9	20.8	22.9
T2	3.1	26.3	13.2	3.0	27.0	11.1	2.5	23.2	18.3
T3	2.0	22.2	21.0	2.1	23.0	19.6	3.8	23.2	17.5
T4	1.9	27.5	9.4	1.9	23.2	21.2	2.3	21.4	21.5
	**TAP_70**			**BC10_70**			**BC20_70**		
	F	C	Fe	F	C	Fe	F	C	Fe
T1	3.2	27.4	10.0	2.3	27.3	8.6	2.5	24.6	13.1
T2	1.8	20.8	23.8	1.8	20.4	26.0	1.5	16.4	30.7
T3	2.3	21.5	21.9	2.3	20.1	22.1	1.3	17.7	27.5
T4	1.8	22.2	16.5	1.7	18.1	25.9	1.1	15.0	35.4

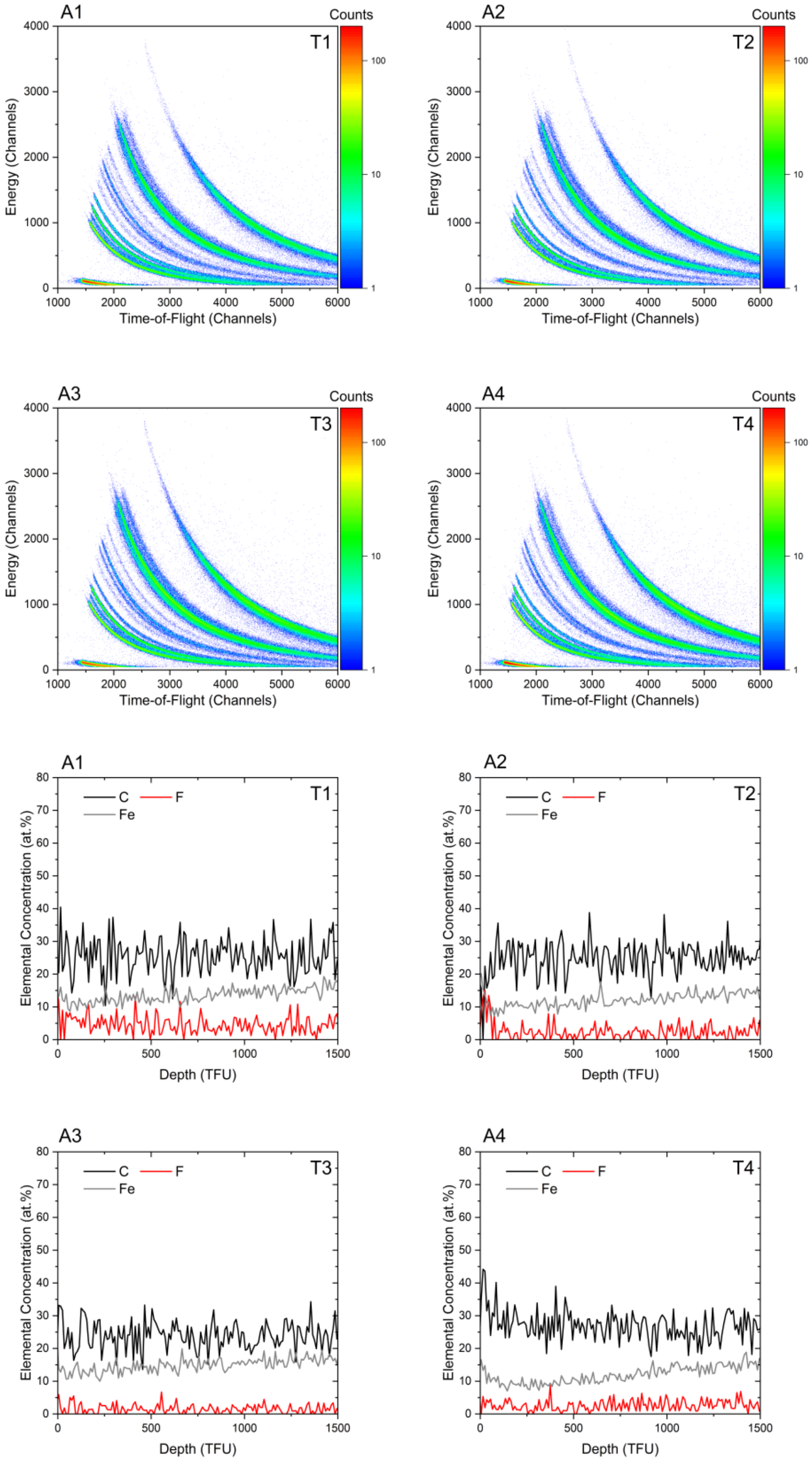

Figure S7: Histograms and depth profiles of TAP at 20°C.

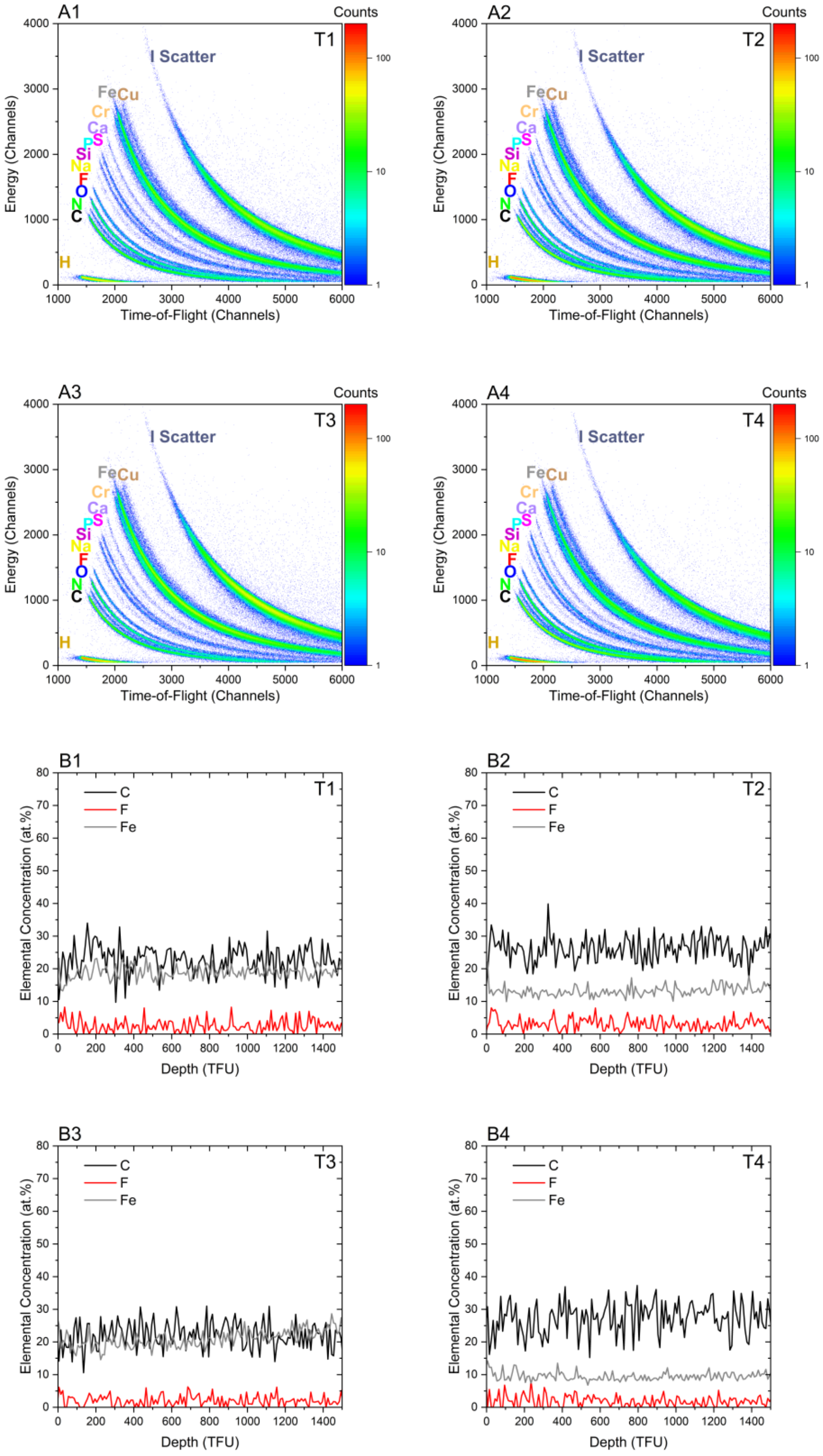

Figure S8: Histograms and depth profiles of TAP at 40°C.

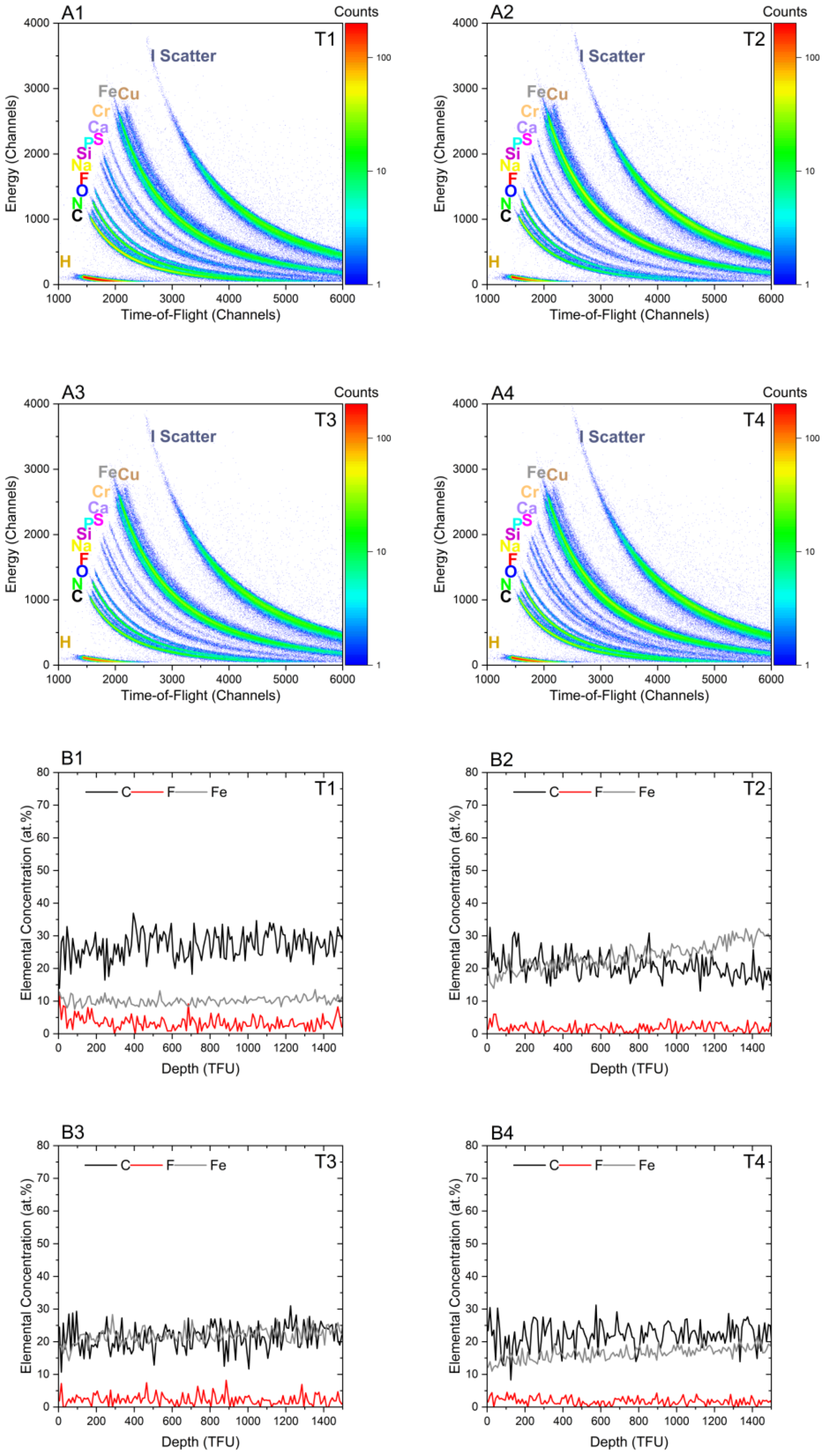

Figure S9: Histograms and depth profiles of TAP at 70°C.

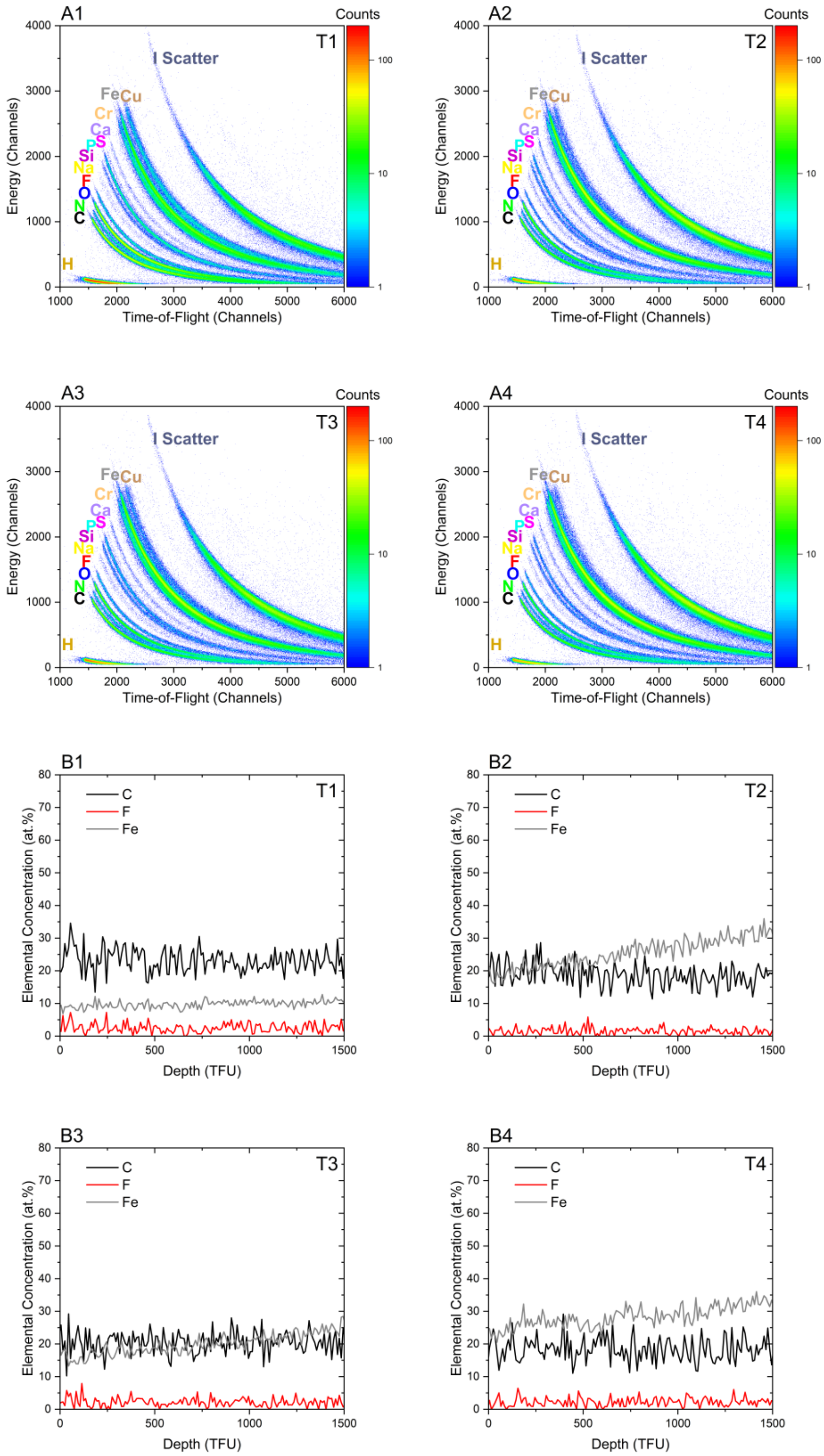

Figure S10: Histograms and depth profiles of MeOH at 20°C.

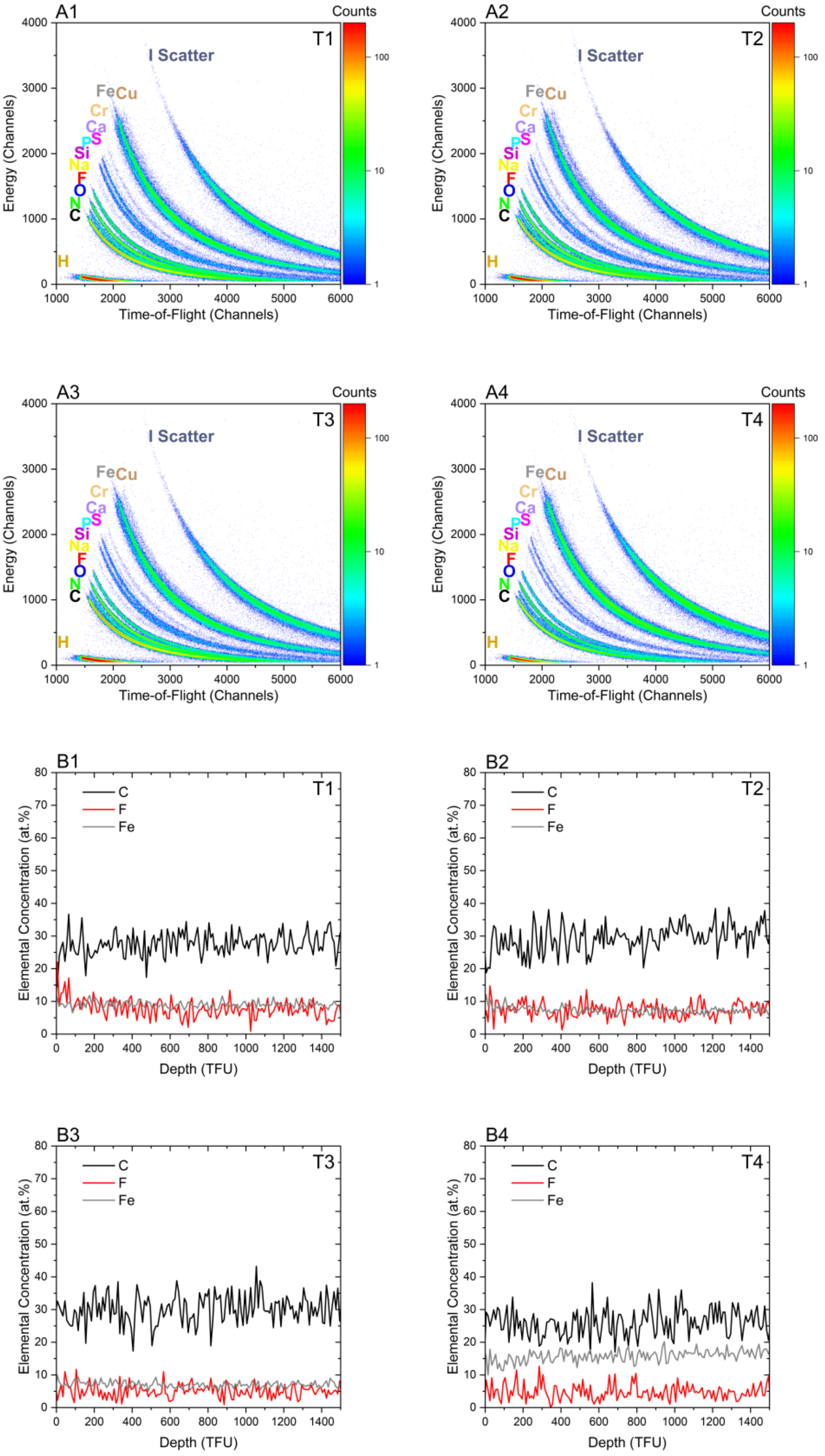
A1
A2
A3
A4
T1
T2
T3
T4
Counts
I Scatter
Fe
Cu
Cr
Ca
P
S
Si
Na
F
O
N
C
H
Energy (Channels)
Time-of-Flight (Channels)
B1
B2
B3
B4
C
F
Fe
Elemental Concentration (at.%)
Depth (TFU)

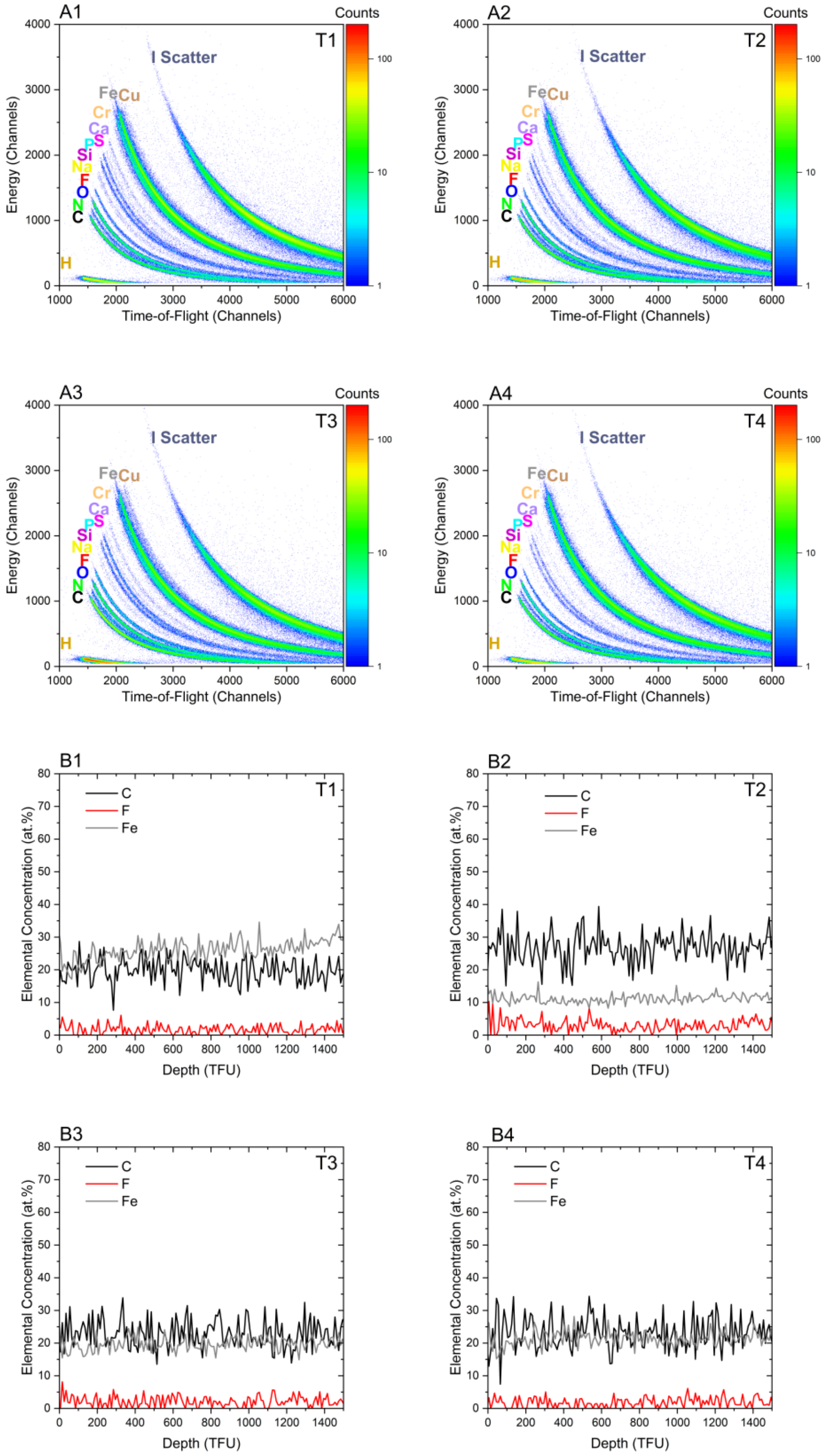

Figure S12: Histograms and depth profiles of BC10 at 40°C.

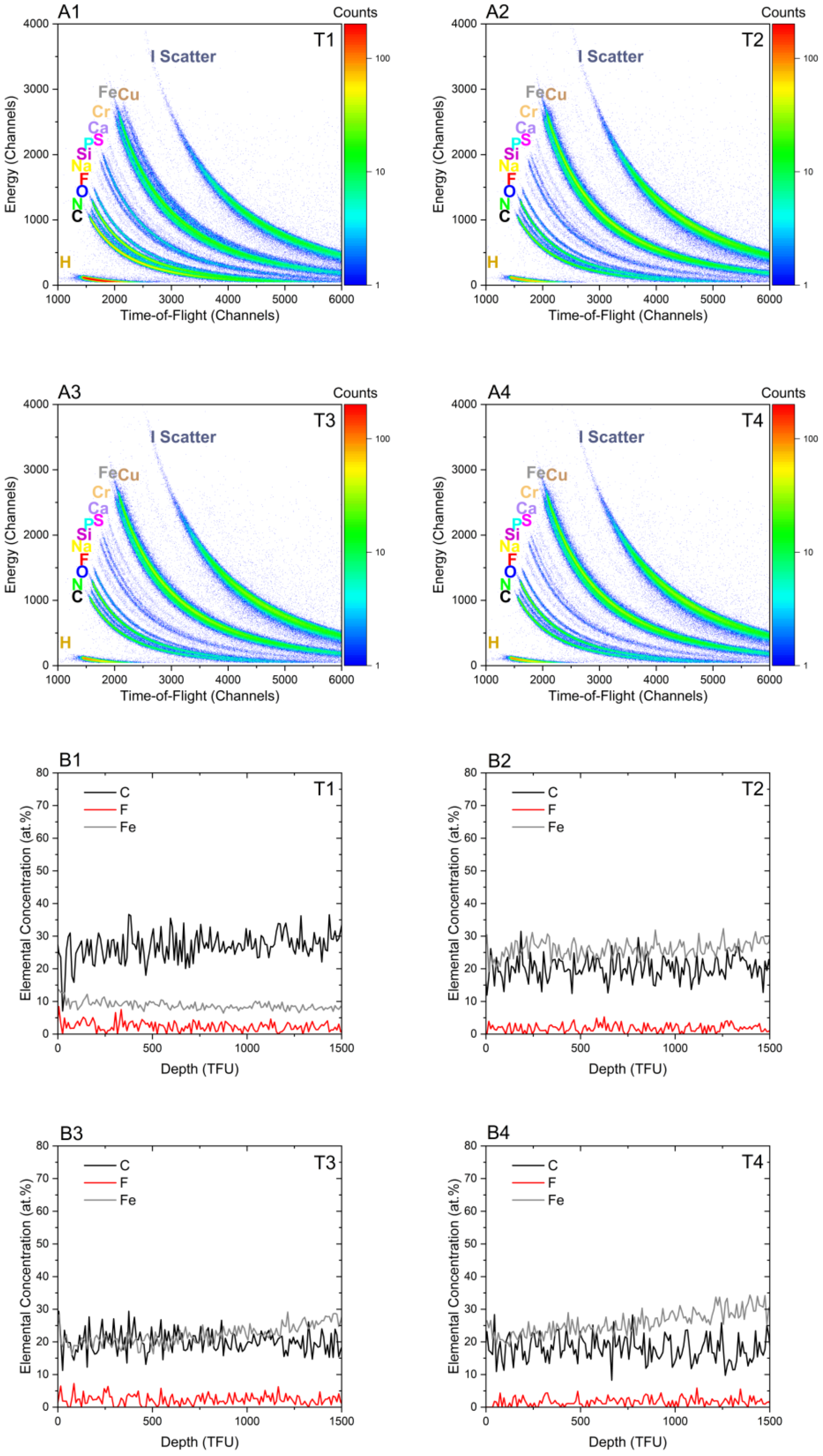

Figure S13: Histograms and depth profiles of BC10 at 70°C.

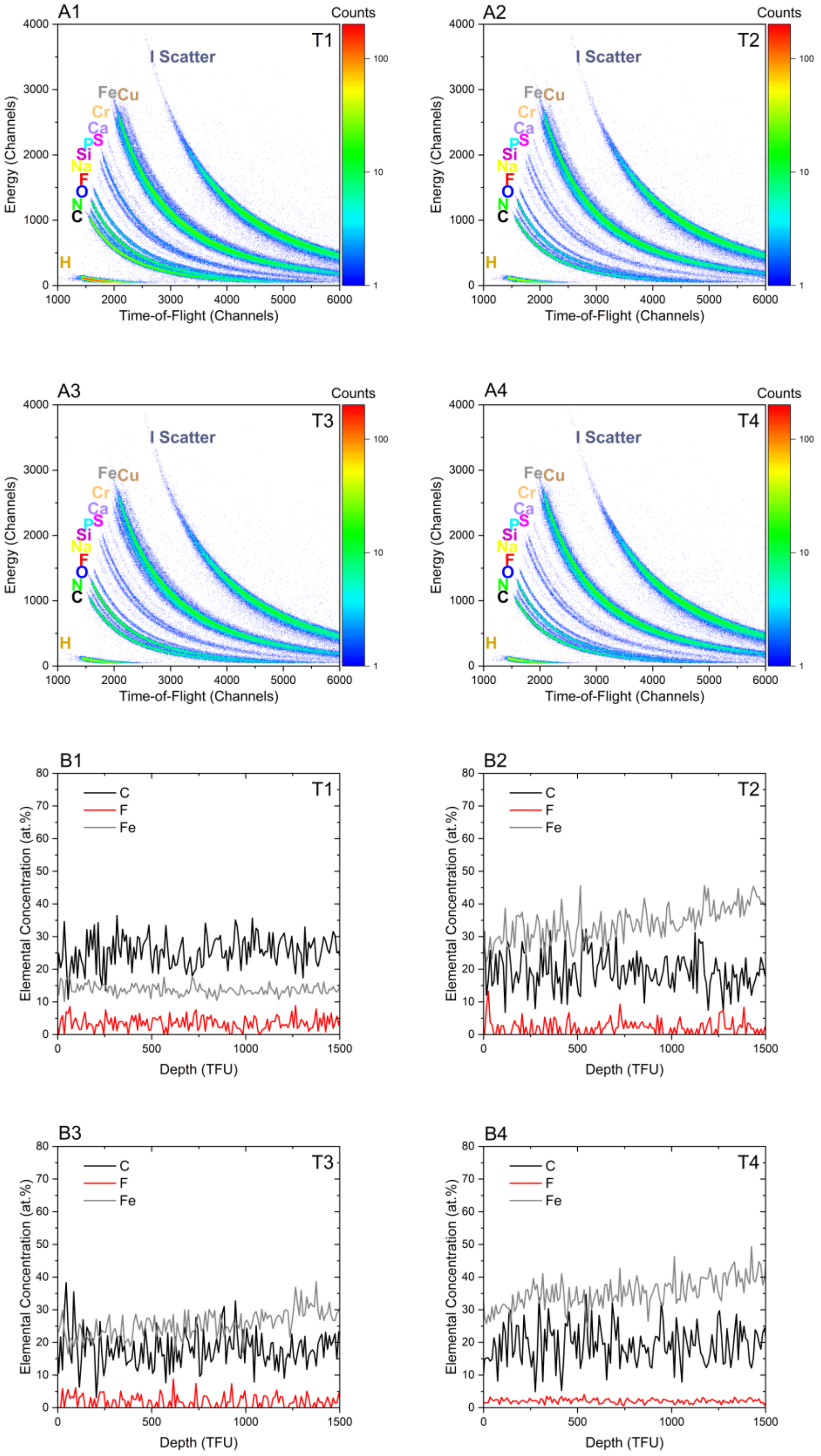

Figure S14: Histograms and depth profiles of BC20 at 20°C.

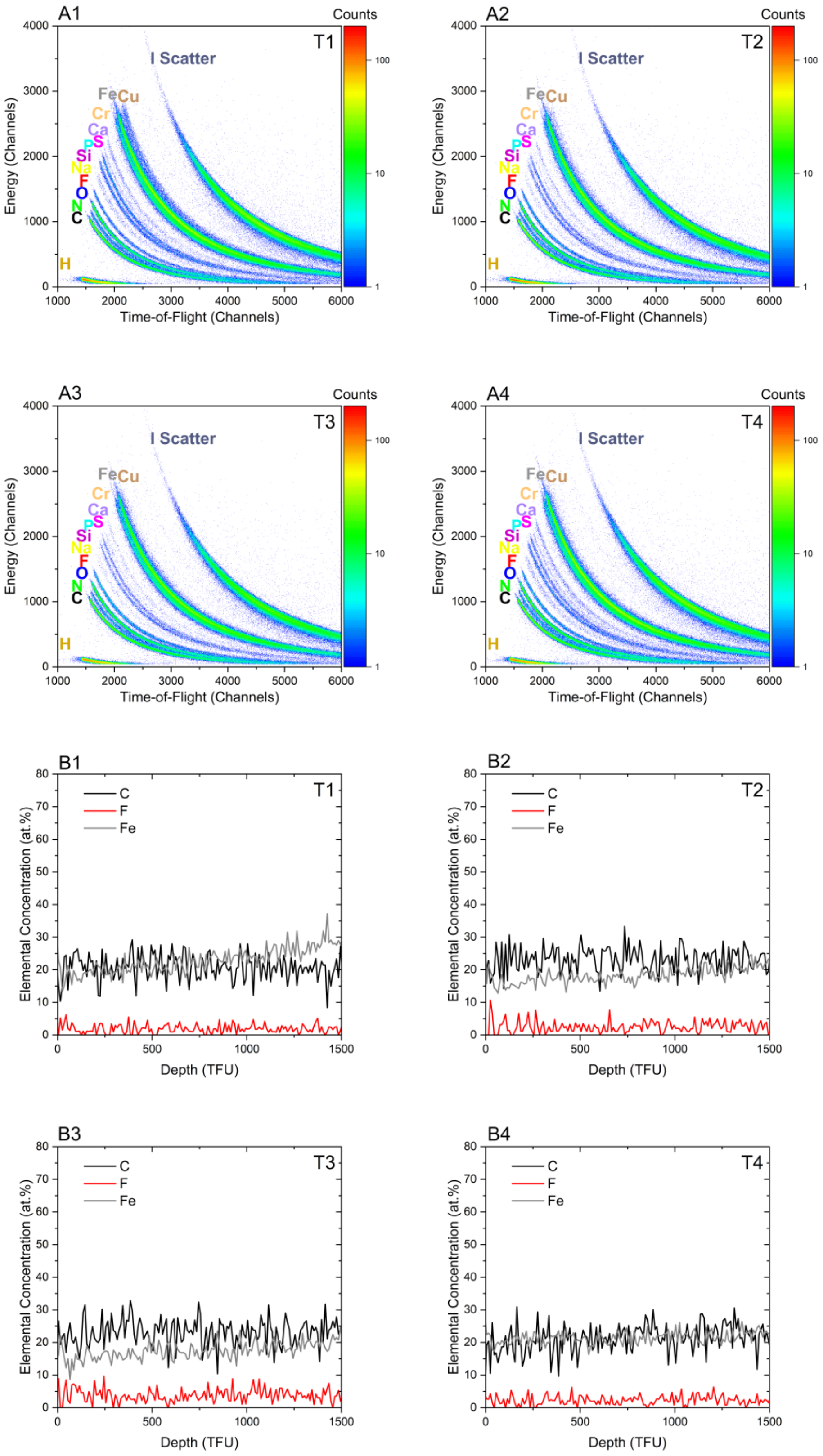

Figure S15: Histograms and depth profiles of BC20 at 40°C.

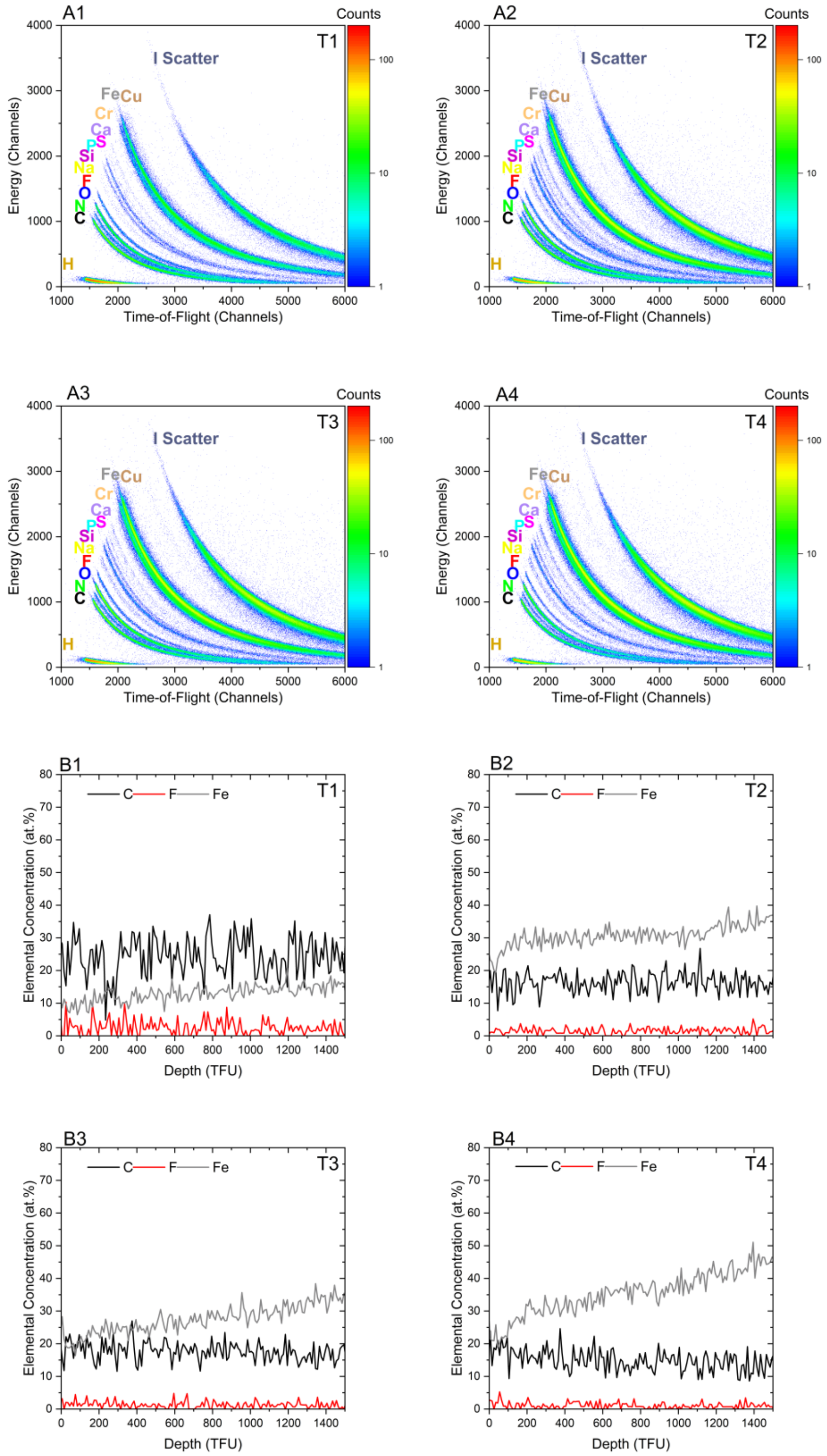

Figure S16: Histograms and depth profiles of BC20 at 70°C.

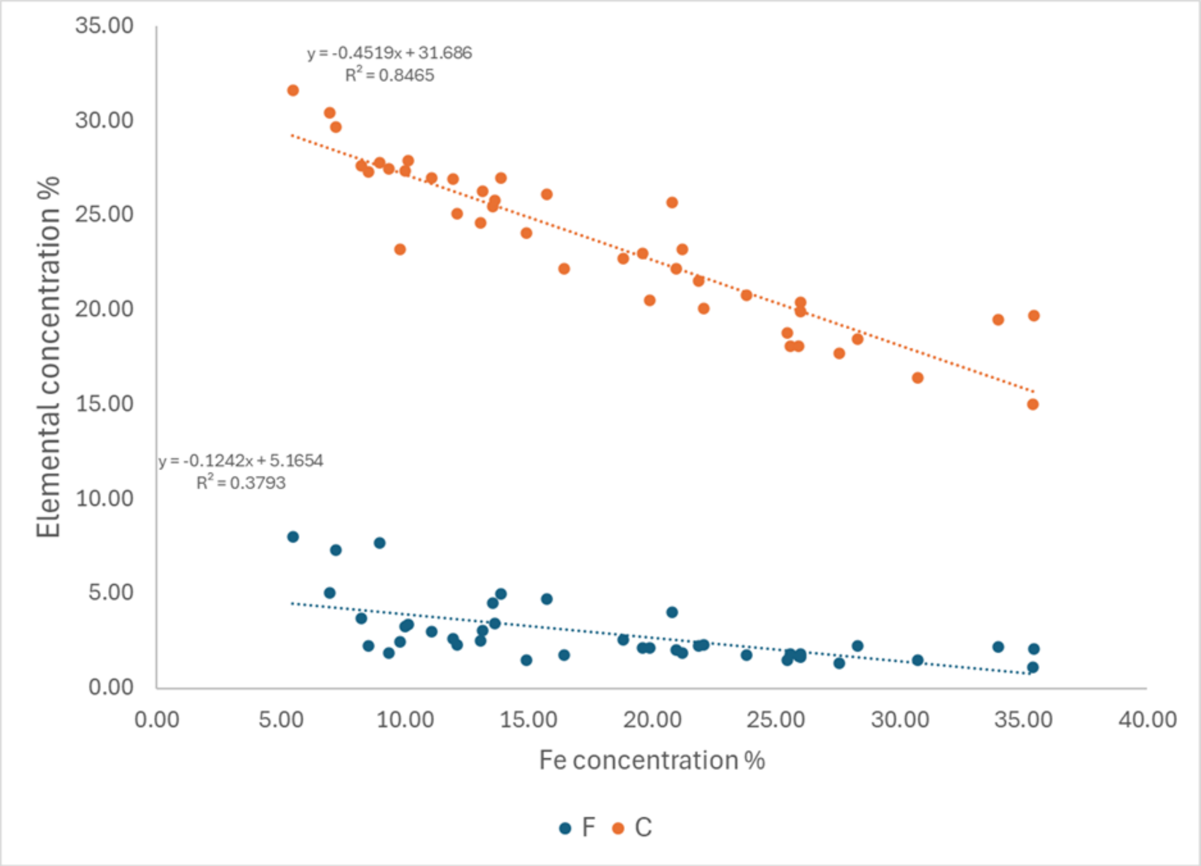

Figure S17: Relationship between surface concentrations of fluorine (F), carbon (C) (y-axis) and iron (Fe) from ToF-ERD measurements. All samples measured with ToF-ERD, including every time step and every soaking solution are included in the Figure.

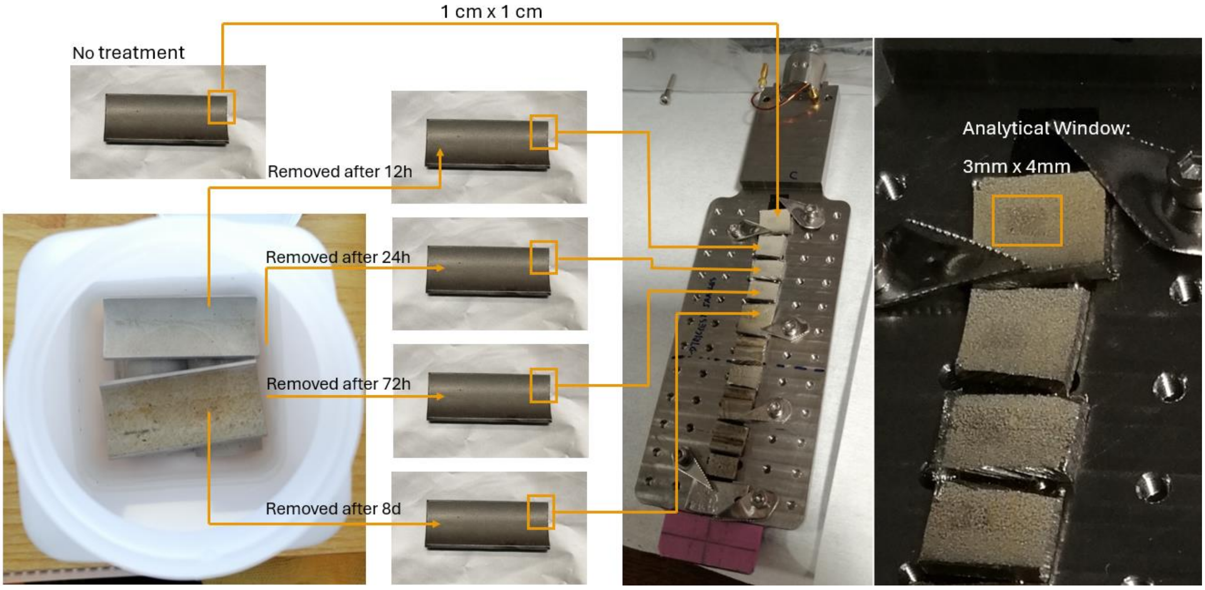

Figure S18: Workflow of preparations for ToF-ERD analysis

S16: Calculation of number of atoms per cm²

The equation for calculating the number of atoms per cm² is given below,

$$n = \left(\frac{elemental\ concentration\ (at.\%) * TFU}{100}\right) * 10^{15}$$

References

1. Shi, Y.; Wright, M.; Sharpe, M. K.; McAleese, C. D.; Polzin, J.-I.; Niu, X.; Zhao, Z.; Morris, S. M.; Bonilla, R. S., Characterization of solar cell passivating contacts using time-of-flight elastic recoil detection analysis. *Applied Physics Letters* **2023,** *123* (26).
2. Julin, J.; Sajavaara, T., Conceptual study of a heavy-ion-ERDA spectrometer for energies below 6 MeV. *Nuclear Instruments and Methods in Physics Research Section B: Beam Interactions with Materials and Atoms* **2017,** *406*, 61-65.
3. Arstila, K.; Julin, J.; Laitinen, M.; Aalto, J.; Konu, T.; Kärkkäinen, S.; Rahkonen, S.; Raunio, M.; Itkonen, J.; Santanen, J.-P., Potku–New analysis software for heavy ion elastic recoil detection analysis. *Nuclear Instruments and Methods in Physics Research Section B: Beam Interactions with Materials and Atoms* **2014,** *331*, 34-41.
4. Kissa, E., *Fluorinated surfactants and repellents*. CRC Press: 2001; Vol. 97.
5. Chang, Q., *Colloid and interface chemistry for water quality control*. Academic Press: 2016.
6. Hubert, M.; Meyn, T.; Hansen, M. C.; Hale, S. E.; Arp, H. P. H., Per-and polyfluoroalkyl substance (PFAS) removal from soil washing water by coagulation and flocculation. *Water Research* **2024,** *249*, 120888.
7. Nguyen, T. M. H.; Bräunig, J.; Thompson, K.; Thompson, J.; Kabiri, S.; Navarro, D. A.; Kookana, R. S.; Grimison, C.; Barnes, C. M.; Higgins, C. P., Influences of chemical properties, soil properties, and solution pH on soil–water partitioning coefficients of per-and polyfluoroalkyl substances (PFASs). *Environmental science & technology* **2020,** *54* (24), 15883-15892.
8. Houtz, E. F.; Sedlak, D. L., Oxidative conversion as a means of detecting precursors to perfluoroalkyl acids in urban runoff. *Environmental science & technology* **2012,** *46* (17), 9342-9349.
